# A comprehensive evaluation of constraining amino acid biosynthesis in compartmented models for metabolic flux analysis

**DOI:** 10.1016/j.meteno.2017.07.001

**Published:** 2017-07-11

**Authors:** Mathias Lehnen, Birgitta E. Ebert, Lars M. Blank

**Affiliations:** iAMB – Institute of Applied Microbiology, ABBt – Aachen Biology and Biotechnology, RWTH Aachen University, Worringer Weg 1, D-52074 Aachen, Germany

**Keywords:** MFA, metabolic flux analysis, MDV, mass distribution vector, Sf, flux solution from an unconstrained model, Sd, flux solution from a fully constrained model, Sd_min_, flux solution from a model with minimal constraints, ACCOA, acetyl-CoA, PYR, pyruvate, SER, serine, GLY, glycine, THR, threonine, ILE, isoleucine, LEU, leucine, TP, TargetP 1.1, WP, WoLF PSORT, ^13^C-metabolic flux analysis, Eukaryotes, Compartmented metabolism, Non-conventional yeast, *S. cerevisiae*, *H. polymorpha*

## Abstract

Recent advances in the availability and applicability of genetic tools for non-conventional yeasts have raised high hopes regarding the industrial applications of such yeasts; however, quantitative physiological data on these yeasts, including intracellular flux distributions, are scarce and have rarely aided in the development of novel yeast applications. The compartmentation of eukaryotic cells adds to model complexity. Model constraints are ideally based on biochemical evidence, which is rarely available for non-conventional yeast and eukaryotic cells. A small-scale model for ^13^C-based metabolic flux analysis of central yeast carbon metabolism was developed that is universally valid and does not depend on localization information regarding amino acid anabolism. The variable compartmental origin of traced metabolites is a feature that allows application of the model to yeasts with uncertain genomic and transcriptional backgrounds. The presented test case includes the baker's yeast *Saccharomyces cerevisiae* and the methylotrophic yeast *Hansenula polymorpha*. Highly similar flux solutions were computed using either a model with undefined pathway localization or a model with constraints based on curated (*S. cerevisiae*) or computationally predicted (*H. polymorpha*) localization information, while false solutions were found with incorrect localization constraints. These results indicate a potentially adverse effect of universally assuming *Saccharomyces-*like constraints on amino acid biosynthesis for non-conventional yeasts and verify the validity of neglecting compartmentation constraints using a small-scale metabolic model. The model was specifically designed to investigate the intracellular metabolism of wild-type yeasts under various growth conditions but is also expected to be useful for computing fluxes of other eukaryotic cells.

## Highlights

• Compartmentation influences computed intracellular fluxes.
• Improper localization constraints potentially produce false flux solutions.
• Minimal compartmentation constraints result in high-quality flux computations.

## 1 Introduction

Knowledge of intracellular reaction rates (fluxes) is crucial to understand how cells metabolize nutrients and how they adapt the fluxes in response to environmental or genetic perturbations. The investigation of intracellular reaction rates has become accessible to a wide range of scientists, rather than exclusively experts, and has been applied to expand detailed knowledge on cellular physiology (Blank et al., 2005, Long and Antoniewicz, 2014, Petersen et al., 2000) as well to guide metabolic engineering (Bartek et al., 2011, Stephanopoulos, 1999, Toya and Shimizu, 2013) and biomedical research (Boghigian et al., 2010). The field of fluxomics has not only diversified but has also matured extensively since its inception, and a wide range of biological questions can be addressed through flux balance analysis (Orth et al., 2010), metabolic flux analysis (MFA) (Bonarius et al., 1996, Vallino and Stephanopoulos, 1990), ^13^C-based MFA (Quek et al., 2009, Weitzel et al., 2013, Wiechert, 2001, Zamboni et al., 2005) or non-stationary MFA (Wiechert and Nöh, 2013). Furthermore, novel methods, such as two-scale (2S)-^13^C-MFA (Martin et al., 2015), are still emerging. Comprehensive frameworks and guidelines are available to simplify and optimize every step of the analysis, including model generation, experimental design, analytical methods, visualization, and statistical evaluation of simulated results (Crown and Antoniewicz, 2013, Droste et al., 2013, Ebert et al., 2012, Nöh et al., 2014, Wiechert et al., 2001).

MFA computes intracellular fluxes by solving a set of metabolite mass balances for an organism in a metabolic steady state. ^13^C-MFA is an extension of this type of analysis based on the use of isotopically enriched carbon substrates. As the incorporation of ^13^C-isotopes into metabolites depends on the flux distribution, tracking the distribution of the ^13^C-isotopes in metabolic intermediates or end products, e.g., proteinogenic amino acids, allows the generation of additional constraints to the metabolite mass balance equations, either as metabolic flux ratios (Sauer et al., 1999) or in the form of isotopomer (*isotope isomer*) balances (Wiechert and de Graaf, 1996). These additional constraints enhance the potential to identify intracellular fluxes, especially for parallel pathways that result in distinct distributions of the tracer in the metabolites. ^13^C-MFA has therefore become a standard tool, especially in the context of research on bacterial metabolism, with isotopomer modeling being the most commonly used method at present. More specifically, the metabolite and isotopomer balances are constrained with the experimentally determined substrate uptake, product formation, and biomass production rates as well as labeling information of metabolites in the form of mass distribution vectors (MDVs). An MDV describes the relative abundance of mass isotopomers differing in the number of included ^13^C-isotopes of a single metabolite or a fragment thereof and are determined by mass spectrometric analyses. The equation system is solved, i.e., the fluxes are computed, by least squares parameter estimation, in which an initial random flux distribution is iteratively adapted to minimize the sum of the squared residuals (SSR) between experimentally and simulated MDVs. A χ^2^-cut-off defines the maximum SSR value for a statistically acceptable flux solution. The solution with the smallest SSR in a sufficient number of minimizations is then assumed to be the optimal flux solution. Flux confidence intervals as a result of a sensitivity analysis indicate the resolvability and thus the robustness of the flux solutions (Antoniewicz et al., 2006).

A review of publications on ^13^C-MFA reveals that few published studies have focused on eukaryotes, with *S. cerevisiae* being the most prominent organism among the investigated eukaryotes. This is partially due to the high scientific and industrial interest in baker's yeast but also to some other extent to the additional modeling challenges arising from the complexity of compartmented organisms. Specifically, the duplication of metabolite pools in distinct compartments and the corresponding parallelization of metabolic pathways increases the solution space and the extent of necessary calculations while decreasing the available constraining information by lumping isotopic labeling data of metabolites from separate compartments in one measurable pool. The issue of modeling compartmentation is usually mentioned in eukaryotic MFA studies but is rarely addressed and resolved in detail (Blank et al., 2005, dos Santos et al., 2003, Förster et al., 2014, Gopalakrishnan and Maranas, 2015). At the core of the pathway parallelization problem is the biosynthesis of proteinogenic amino acids (Förster et al., 2014), whose labeling patterns are usually measured and used as proxy for the labeling of free intracellular metabolites (Szyperski, 1995). The crux in eukaryotic cells is the incomplete knowledge regarding the compartmental origin of precursor metabolites. Even in well-described organisms such as *S. cerevisiae*, these knowledge gaps exist, with alanine biosynthesis being one example (Blank et al., 2005, Buescher et al., 2015). Although defined as catalytically inactive in *S. cerevisiae*, the cytosolic equivalent to the mitochondrial alanine transaminase is genetically available and only transcriptionally regulated. In non-conventional yeasts or other poorly characterized organisms, this problem expands to full uncertainty regarding the existence and activity of enzymes and pathways in any modeled compartment and does not only concern alanine, but theoretically any amino acid that originates from a central metabolite present in more than one compartment. In a modeling context, this indicates that metabolic pathways cannot always be confined to one of multiple compartments, nor is sufficient information available to define a specific ratio between parallel pathways. Thus, we address here the necessity of creating a model with an option of undefined compartmental origin of biosynthetic precursors, especially for eukaryotic systems that are not as well characterized as *S. cerevisiae.* For model validation, we chose the model organism *S. cerevisiae* CEN.PK and the less well-characterized yeast *Hansenula polymorpha*, for which an annotated genome sequence and physiological data were available.

## 2 Materials and methods

### 2.1 Strains and culture conditions

*S. cerevisiae* CEN.PK 113 7D (European *S. cerevisiae* Archive for Functional Analysis, http://www.uni-frankfurt.de/fb15/mikro/euroscarf/) and *H. polymorpha* (*Pichia angusta*) CLIB 421 (Collection de levures d′intérêrt biotechnologique, http://www.inra.fr/Internet/Produits/clib/) were used in all experiments. Growth experiments were conducted in 1.3 L shake flasks filled with 50 mL Verduyn medium containing per liter, 5 g (NH_4_)_2_SO_4_, 3 g KH_2_PO_4_, 0.5 g MgSO_4_·7H_2_O, 4.5 mg ZnSO_4_·7H_2_O, 0.3 mg CoCl_2_·6H_2_O, 1.0 mg MnCl_2_·4H_2_O, 0.3 mg CuSO_4_·5H_2_O, 4.5 mg CaCl_2_·2H_2_O, 3.0 mg FeSO_4_·7H_2_O, 0.4 mg NaMoO_4_·2H_2_O, 1.0 mg H_3_BO_3_, 0.1 g KI, 15 mg EDTA, 0.05 mg biotin, 1.0 mg calcium pantothenate, 1.0 mg nicotinic acid, 25 mg inositol, 1.0 mg pyridoxine, 0.2 mg p-aminobenzoic acid, and 1.0 mg thiamine (Verduyn et al., 1992). The medium was supplemented with 5 g/L glucose and buffered with 100 mM potassium hydrogen phthalate. The pH of the medium was adjusted to 5. The shake flasks provided a closed system equipped with O_2_- and CO_2_-sensors (BlueSens gas sensor GmbH, Herten, Germany) and an air-tight sample port, and were shaken at 130 rpm with an amplitude of 30 mm. ^13^C-tracer experiments were performed with a mixture of 80% 1-^13^C-glucose and 20% U-^13^C-glucose (both purchased from Sigma-Aldrich, Steinheim, Germany, with 99 atom-% purity), reported to have a high potential for resolving the network of central carbon metabolism at a reasonable prize per experiment (Zamboni et al., 2009). The main cultures were inoculated to a starting optical density (OD_600_) of 0.05 from precultures grown in the same minimal medium, but supplemented with naturally labeled glucose after cells were harvested and washed with 0.9% NaCl solution. Precultures were grown in 100 mL shake flasks at a shaking frequency of 200 rpm at 30 °C and 40 °C for *S. cerevisiae* and *H. polymorpha*, respectively. Samples of the main culture were taken by connecting a syringe to the sample port, opening the sealing clamp, and flushing the sampling line with air before creating a vacuum with the syringe and allowing approximately 1 mL to flow into the sample tube. The sampling line was flushed with air before the sealing clamp was closed to minimize disturbances of the closed headspace and corresponding gas analysis. After the cultures reached stationary phase, the headspace analysis was continued for several hours to verify the tightness of the system by stable gas analysis signals.

### 2.2 Physiological data acquisition and processing

Immediately after sampling, the OD_600_ was measured, and the samples were centrifuged at 13,000 rpm for 5 min in a tabletop centrifuge. The biomass pellet was separated from the supernatant, and both fractions were stored at −20 °C until further analysis. The supernatant was used to determine the concentrations of glucose and the excreted metabolites ethanol, glycerol, and acetate. The metabolites were separated on an Aminex HPX-87H column (Bio-Rad, Hercules, CA, USA) at 60 °C with a flow rate of 0.8 mL/min of 5 mM H_2_SO_4_. Glucose, ethanol, and glycerol were detected on a Shodex RI-101 detector, and acetate was detected at a wavelength of 210 nm in a variable wavelength detector of an UltiMate 3000 HPLC system (Dionex, Sunnyvale, CA, USA).

The cell dry weight (CDW) of samples was calculated from the measured OD_600_ and a calibration curve, which was recorded for both yeasts under corresponding experimental conditions. The correlation curves were generated by weighing the biomass from duplicates of 10 mL culture samples with five different OD_600_ values ranging from 0.5 to 6. The samples were cooled on ice before centrifugation in glass tubes at 3500 rpm for 20 min in a Heraeus Megafuge 16 R (Thermo Scientific, Waltham, MA, USA). The supernatant was discarded, and biomass pellets were washed with water and centrifuged again. Finally, the supernatant was discarded again and biomass pellets were dried at 60 °C for 48 h before weighing.

The off-gas was measured using BCP-O_2_ and BCP-CO_2_ sensors connected to a BACCom12 communication box using the FermVis software (BlueSens gas sensor GmbH, Herten, Germany). The signals were obtained in units vol% (H) and transformed into molar concentrations as described by Heyland et al. (2009) using Eqs. (1), (2), in which V_R_ is the real molar volume, and V_m_ is the volume of one mole of ideal gas.

(1) $${\text{c}}_{{\text{CO}}_{\text{2}}\text{,}{\text{O}}_{\text{2}}}\text{ =}\;\frac{\text{n}}{{\text{V}}_{\text{g}}}\;\text{=}\;\frac{\text{H ∙}{\text{V}}_{\text{g}}}{{\text{V}}_{\text{R}}}\text{∙}\frac{\text{1}}{{\text{V}}_{\text{g}}}\text{[}\text{mM}\text{]}$$

(2) $${\text{V}}_{\text{R}\;}\text{=}\;\frac{{\text{V}}_{\text{m}}\text{∙}\text{1.013}\text{∙}\text{T}}{\text{p}\text{∙}\text{273.15}}\text{[L}{\text{mol}}^{\text{-1}}\text{]}$$

Exponential growth rate (µ), uptake rates, and production rates (r) were calculated by a simultaneous nonlinear fit of the time-dependent concentration (c) changes using Eqs. (3), (4), (5), (6), (7), in which x_0_, s_0_, and p_0_ are the biomass, substrate, and product concentrations, respectively, at the beginning of the experiment; x_t_ is the biomass at time point t, and Y_SX_ and Y_PX_ are the substrate and product yields on biomass, respectively. The equations were solved with SigmaPlot Version 12.5 (Systat Software, Inc., San Jose, CA, USA). For experiments in which the formation of glycerol, acetate or ethanol was not observed, the corresponding equations were excluded from the fit.

(3) $$\text{μ}\text{ =}\;\text{ln(}{\text{x}}_{\text{t}}\text{ -}{\text{ x}}_{\text{0}}\text{)}/\text{∆t}\text{[}{\text{h}}^{\text{-1}}\text{]}$$

(4) $${\text{c}}_{\text{glucose,}{\text{O}}_{\text{2}}\;}\text{=}\;{\text{s}}_{\text{0}}\text{ -}{\text{ Y}}_{\text{SX}}\text{∙}\left({\text{x}}_{\text{0}}\text{∙}{\text{e}}^{\text{μ∙t}}\text{ -}{\text{ x}}_{\text{0}}\right)\text{[}\text{mM}\text{]}$$

(5) $${\text{c}}_{\text{glycerol, acetate,}\text{ethanol,}{\text{CO}}_{\text{2}}\;}\text{=}\;{\text{p}}_{\text{0}}\text{ +}\;{\text{Y}}_{\text{PX}}\text{∙}\left({\text{x}}_{\text{0}}\text{∙}{\text{e}}^{\text{μ∙t}\;}\text{-}{\text{ x}}_{\text{0}}\right)\text{[}\text{mM}\text{]}$$

(6) $${\text{r}}_{\text{glucose,}{\text{O}}_{\text{2}}\;}\text{=}{\text{ Y}}_{\text{SX}}\text{∙μ}\text{[}\text{mmol}{\text{g}}^{\text{-1}}{\text{h}}^{\text{-1}}\text{]}$$

(7) $${\text{r}}_{\text{glycerol,}{\text{acetate, ethanol, CO}}_{\text{2}}\;}\text{=}{\text{ Y}}_{\text{PX}}\text{∙μ}\text{[}\text{mmol}{\text{g}}^{\text{-1}}{\text{h}}^{\text{-1}}\text{]}$$

The calculated CO_2_ production rate was not used as an input constraint for ^13^C-MFA because the CO_2_ sensor was calibrated for unlabeled carbon and was therefore insensitive to ^13^C-labeled CO_2_. Specifically, due to the calibration of the sensors, only 6% of ^13^CO_2_ was detected. A pre-simulation correction of this rate was not feasible because the labeling of CO_2_ is not necessarily equal to the total labeling of substrate carbon, but rather depends on the actual flux distribution. Once a flux distribution was found, the simulated CO_2_ labeling was used to correct the original CO_2_ rate, and this rate was used as a quality indicator for the obtained flux solution. The corrected CO_2_ production rates (r_CO2,cor_) were calculated from the measured rates (r_CO2_) and the simulated fraction of unlabeled and labeled CO_2_ (f_m+0_ and f_m+1_, respectively) derived from the best flux solution using Eq. (8).

(8) $${\text{r}}_{{\text{CO}}_{\text{2}}\text{,cor}}\text{ =}\;\frac{{\text{r}}_{{\text{CO}}_{\text{2}}}}{{\text{f}}_{\text{m}\text{ +}\;\text{0}\;}\text{+}\;\text{0.06}\text{ ×}{\text{ f}}_{\text{m}\text{ +}\text{ 1}}}$$

### 2.3 ^13^C-labeling analysis

Biomass that was sampled during the mid-exponential growth phase was used to determine the MDVs of proteinogenic amino acids. Approximately 0.3 mg of biomass was resuspended in 150 µL of a 6 M HCl solution, then hydrolyzed at 105 °C for 6 h and dried overnight. The dried hydrolysate was resuspended in 30 µL of acetonitrile and 30 µL of N-methyl-N-*tert*-butyldimethylsilyl-trifluoroacetamide (MBDS-TFA; CS-Chromatographie Service GmbH, Langerwehe, Germany), followed by incubation at 85 °C for 1 h to allow derivatization. The derivatized samples were analyzed on a gas chromatography-mass spectrometry (GC-MS) single quadrupole system consisting of a TRACE™ GC Ultra and an ISQ single quadrupole MS with electron impact ionization (Thermo Fisher Scientific, Waltham, MA, USA) equipped with a TraceGOLD TG-5SilMS fused silica column (length, 15 m; inner diameter, 0.25 mm; film thickness, 0.25 µm). The GC-MS was operated as described by Schmitz et al. (2017) with a constant gas flow rate of 1 mL/min of helium and a split ratio of 1/15. The injector temperature was set to 270 °C, and the column oven program comprised an initial temperature of 140 °C for 1 min and a temperature ramp with a rate of 10 °C/min to a final temperature of 310 °C with a hold time of 1 min.

The raw GC-MS data were transformed into MDVs and corrected for the influence of unlabeled biomass from the inoculum and the natural abundance of heavy isotopes using iMS2Flux (Poskar et al., 2012), as described by Schmitz et al. (2017).

### 2.4 ^13^C-MFA method

OpenFLUX was used to compute the intracellular fluxes (Quek and Nielsen, 2014, Quek et al., 2009). The software generated mass and isotopomer balances from a model in text format, which defined the reaction stoichiometry and carbon atom transitions of central carbon metabolism, precursor drains for biomass synthesis, and lumped reactions of amino acid biosynthesis, which were used to infer amino acid labeling from the modeled central metabolites. All metabolic reactions were defined as unidirectional (F), and physiologically reversible reactions were split into a forward (FR) and reverse (R) reactions. Biomass formation was approximated by a set of equations that break down all biomass-building reactions (B), such as fatty acid, protein, and nucleic acid production, into several reactions that drained the respective precursors from the modeled network. The demand of precursors for biomass synthesis was derived from published yeast biomass composition data (Christen and Sauer, 2011), but instead of individual drains of cytosolic and mitochondrial acetyl-CoA, pyruvate, and oxaloacetate, only one overall drain was used for each metabolite. Detailed pathways for proteinogenic amino acid synthesis were excluded from the network of mass balances. Instead, pathways of amino acid synthesis starting at the central precursors were summarized in one or two reactions per amino acid with the corresponding atom transitions (S/SF), which allowed the computation of amino acid labeling from the labeling of the metabolite precursors. For amino acids that originate from precursors present in both the cytosol and mitochondria, parallel reactions were defined that drained either the cytosolic or mitochondrial pool for their synthesis. In this way, localization constraints could easily be set in the model when conclusive localization information was available. Otherwise, the ratio of the fluxes through these parallel pathways was left unconstrained and was simulated during parameter estimation. More specifically, this was achieved by defining the parallel pathways as SF reactions in the OpenFLUX model. This reaction type indicates that the imaginary fluxes through the parallel reactions add up to unity. In cases of a known location of this segment, the activity (termed basis in OpenFLUX) of the correctly located reaction was set to one and the other to zero. Otherwise, one of the two complementary reactions was defined as a so-called free flux (see the OpenFLUX manual for a detailed definition of flux basis and free fluxes), meaning that the ratio can vary freely during the simulation.

To simulate the label distribution in the metabolic network, metabolites, proteinogenic amino acids, and the carbon atom transitions of the reactions were defined as described by Wiechert and de Graaf (1997). Information regarding the atom transitions was obtained from the BioCyc knowledgebase for *S. cerevisiae* ATCC 18824 (Caspi et al., 2012). The first and second positions of the carbon atom strings of all amino acids were assigned to the carboxyl and amino carbons.

To determine the experimental error of MDVs, the uncertainty of the GC-MS measurements of each amino acid fragment was used as the basis and corrected for variations between measurements from samples taken in short time intervals during the mid-exponential phase of the same culture. For every mass whose relative abundance showed a higher deviation between two consecutive samples than the measurement error of the GC-MS, this absolute deviation was used as error measure.

The model was constrained with glucose and oxygen uptake, byproduct secretion rates, and precursor drains into biomass. The glucose uptake rate was normalized to 1000, while all other constraining rates were scaled accordingly. These rates were defined in a separate MATLAB file (‘preSolverScript.m’) that contained a matrix with the lower and upper boundaries of the respective rates. In the presented model, one uptake reaction for each of the glucose isotopomers (1-^13^C-glucose and U-^13^C-glucose) was included with an undefined ratio. In this manner, the model could compensate for possible small deviations from the intended ratio of the two isotopomers, which may occur due to weighing errors or inaccurate declaration of ^13^C-enrichment. As OpenFLUX requires a flux basis for starting the parameter estimation, the biomass-building drain of glycine was fixed in every model. The complete models, including MDVs, measurement errors, and flux ranges of extracellular reaction rates defined in the preSolverScript files, are included in the supplement.

All computations were performed in MATLAB 2010b (The MathWorks, Inc., MA, USA). Java 7 (Sun Microsystems, Santa Clara, CA, USA) was used to convert the model in text file format into the respective MATLAB files. The resulting flux distributions were visualized on metabolic pathway maps created with Omix® (Droste et al., 2013).

### 2.5 Methods for pathway localization analysis

The *Saccharomyces* Genome Database (www.yeastgenome.org) (Cherry et al., 2012) was used to obtain curated information regarding the compartmental localization of gene products in *S. cerevisiae.* The focus was specifically concentrated on the very reactions in any pathway segment that introduce carbon atoms from one of the central network intermediates with separate pools in both compartments. To investigate pathway localization in *H. polymorpha*, the protein sequences from *S. cerevisiae*, which correspond to essential enzymes in amino acid biosynthesis pathways were subjected to BLAST searches against the protein sequences derived from the *H. polymorpha* genome sequence available from the homepage of the Joint Genome Institute (JGI) (http://genome.jgi.doe.gov/Hanpo2/Hanpo2.home.html, accessed in April 2016). Hits from the BLASTP analysis and annotation information from the JGI database were compared. Resulting BLASTP hits were computationally analyzed with TARGETP 1.1 (TP) (Emanuelsson et al., 2007) and WoLF PSORT (WP) (Horton et al., 2007). Furthermore, the JGI database links the protein sequences with enzyme activities and pathway affiliation. These results were further used to generate the best guess regarding pathway localization in both investigated datasets.

## 3 Results and discussion

### 3.1 Model development considering uncertainties in compartmental origin of amino acids

For metabolic flux analysis, a model was developed that described the central carbon metabolism, including glycolysis, the pentose phosphate pathway (PPP), and the tricarboxylic acid (TCA) cycle, with approximately 50 reactions and linked biomass production to twelve intermediates of this central network. A CO_2_ exchange reaction according to Leighty and Antoniewicz (2012) was used to compensate for dilution of the ^13^C label by naturally labeled CO_2_ contained in the headspace of the shake flask at the beginning of the experiment. A similar compensation was applied for methyltetrahydrofuran (MTHF), allowing label adjustment. A simplified respiratory chain reaction was used to allow reoxidation of NADH and balancing of O_2_. The model was compartmented into cytosol and mitochondrion and featured transport reactions between the compartments for isocitrate, malate, acetyl-CoA (ACCOA), pyruvate (PYR), and oxaloacetate (OAA). The amino acids serine (SER), glycine (GLY), and threonine (THR) were exceptions in the model, as they contributed measured MDVs and were featured as mass-balanced intermediates of the modeled network. These exceptions were made because of the complex interactions among the three amino acids and with the rest of the network. Furthermore, the model definition allowed to simulate the origin of each amino acid independently and uncoupled from other amino acids stemming from the same precursor, e.g., alanine and leucine, which share the common precursor pyruvate.

The model reactions for valine (VAL), leucine (LEU), and isoleucine (ILE) biosynthesis shown in Table 1 will be used as an example of the model structure.

**Table 1:** Modeling of amino acid biosynthesis. Shown is an excerpt of the metabolic model (Supplementary Table S1) with a consecutive number of reaction IDs and abbreviated reaction equations. The indices ‘mit’ and ‘cyt’ indicate mitochondrial or cytosolic compartmentation, respectively. In the case of isoleucine, the indices ‘o′ and ‘t′ indicate ILE originating from the pathways incorporating OAA and THR, respectively.

**Reaction ID**	**Reaction Equation**
**r118**	PYR_mit_ + PYR_mit_ = VAL + CO_2_
**r119**	PYR_cyt_ + PYR_cyt_ = VAL + CO_2_
**r120**	PYR_cyt_ + OAA_cyt_ = ILE_o_ + CO_2_
**r121**	PYR_mit_ + OAA_mit_ = ILE_o_ + CO_2_
**r122**	PYR_cyt_ + THR = ILE_t_ + CO_2_
**r123**	PYR_mit_ + THR = ILE_t_ + CO_2_
**r124**	ILE_o_ = ILE
**r125**	ILE_t_ = ILE
**r126**	PYR_cyt_ + PYR_cyt_ = ISV + CO_2_
**r127**	PYR_mit_ + PYR_mit_ = ISV + CO_2_
**r128**	ISV + ACCOA_mit_ = LEU + CO_2_
**r129**	ISV + ACCOA_cyt_ = LEU + CO_2_

To independently simulate the labeling patterns of leucine and valine, which share the common precursor 2-oxoisovalerate (ISV), the synthesis pathways of these two amino acids had to be uncoupled. To this end, leucine synthesis was defined by two reactions, pyruvate condensation (r128) and acetylation (r129), to account for a possible exchange of the intermediate 2-oxoisovalerate between compartments. In contrast, valine synthesis was aggregated into one reaction, thereby skipping the intermediate 2-oxoisovalerate (Table 1) and, hence, avoiding interference of the labeling simulation of valine and leucine. A similar simplification was not feasible in the cases of threonine and isoleucine because threonine could not be directly substituted by a single central carbon metabolite, as it is derived from two alternative pathways, i.e., the amination of oxaloacetate and the condensation of glycine and acetaldehyde. Consequently, to uncouple threonine and isoleucine biosynthesis, an additional shortcut pathway that directly synthesized isoleucine from oxaloacetate (r120 and r121 in Table 1) was introduced, while the original pathway (r122 and r123) was maintained to allow partial isoleucine synthesis from threonine derived from glycine. As only the average labeling of both amino acid pools could be measured, reactions r124 and r125 combined all isoleucine synthesis pathways into this average pool. Defining the subspecies ILEo and ILEt was necessary because OpenFLUX did not allow free variation of the four possible isoleucine synthesis reactions (see the Materials and methods section for details).

### 3.2 Computational predictions of amino acid biosynthesis compartmentation

Curated information on the localization of amino acid biosynthesis for *S. cerevisiae* was obtained from the *Saccharomyces* Genome Database. Eleven genes that contribute to nine anabolic pathways or pathway segments that link amino acids to precursors with separate cytosolic and mitochondrial pools were identified. The localization in the non-conventional yeast *H. polymorpha* was not reported and was inferred with the computational localization analysis tool WoLF PSORT and TargetP based on the protein sequences of the eleven identified genes. The comparison with protein annotations from the JGI database confirmed the BLASTP results and yielded no further protein entities suitable for analysis. The computational prediction of the most likely compartmental pathway localization was used to generate a pathway localization pattern for *H. polymorpha.* A summary of genes and proteins with the results of the computational analysis of compartmental localization is provided in Table 2.

**Table 2:** Results of the computational analysis regarding the mitochondrial (mit) or cytosolic (cyt) localization of key enzymes involved in amino acid synthesis. In cases of multiple BLASTP hits, the computed localizations of individual hits are separated by a ‘+’ symbol, and results from the prediction tools WolfPsort (WP) and TargetP (TP) are separated by a slash (/). Instances where localization could not be predicted with certainty are indicated by a question mark (?).

**Biosynthesis Pathway**	**Gene**	**Protein**	**Curated*****S. cerevisiae*****localization**	**Predicted*****S. cerevisiae*****localization (WP)/ (TP)**	**Predicted localization of*****H. polymorpha*****BLASTP hits (WP)/ (TP)**
**Valine/Leucine/ Isoleucine**	ILV2	**Acetolactate synthase**	mit	mit/mit	mit/mit
**Isoleucine**	ILV1	**Threonine deaminase**	mit	mit/?	?+?/mit+mit
**Leucine**	LEU4	**α-Isopropylmalate synthase**	?	cyt/cyt	?+cyt/mit+cyt
	LEU9	**LEU4p isoenzyme**	mit	cyt/mit	?+cyt/mit+cyt
**Aspartate/Threonine/ Methionine**	AAT1	**Aspartate aminotransferase**	mit	mit/mit	mit+cyt/mit+cyt
	AAT2	**AAT1p isoenzyme**	cyt	cyt/cyt	mit+cyt/mit+cyt
**Threonine/Methionine**	HOM3	**Aspartate kinase**	cyt	cyt/?	no hit
**Lysine**	LYS21	**Homocitrate synthase**	?	cyt/cyt	cyt+cyt/cyt+cyt
	LYS20	**LYS21p isoenzyme**	cyt	cyt/cyt	cyt+cyt/cyt+cyt
**Alanine**	ALT1	**Alanine transaminase**	mit	mit/mit	?/mit
	ALT2	**ALT1p isoenzyme (inactive)**	cyt	cyt/cyt	?/mit

Based on the curated localization information provided in Table 2, valine, leucine, isoleucine, and alanine biosynthesis in the *S. cerevisiae* model was constrained to the mitochondria, while lysine biosynthesis was constrained to the cytosol. For lysine biosynthesis, this concerned only the carbon atoms stemming from acetyl-CoA, as the second precursor α-ketoglutarate (OGA) was exclusively located in the mitochondria. The uncertainties in the localization of leucine and lysine biosynthesis were neglected, as it is has been shown by Gombert et al. (2001) that acetyl-CoA involved in leucine biosynthesis is of mitochondrial origin, while the acetyl-CoA involved in lysine biosynthesis is of cytosolic origin. Furthermore, there were several instances where prediction was not possible, indicated by a question mark (?) in Table 2. The prediction results for *H. polymorpha* BLASTP hits were consistently mitochondrial for valine and alanine biosynthesis and consistently cytosolic for lysine biosynthesis. In the cases of leucine and isoleucine biosynthesis, inconsistent results were neglected, as such inconsistencies were neglected for *S. cerevisiae* as well, and localizations were assumed to be mitochondrial. The localization of aspartate, threonine, and methionine biosynthesis was left variable based on parallel cytosolic and mitochondrial prediction results. This resulted in an overall pathway map of *H. polymorpha* that was identical to that of *S. cerevisiae*. The final compartmentation constraints are indicated in the corresponding models in the supplement.

### 3.3 Quantitative physiology of *S. cerevisiae* and *H. polymorpha*

**Fig. 1:**
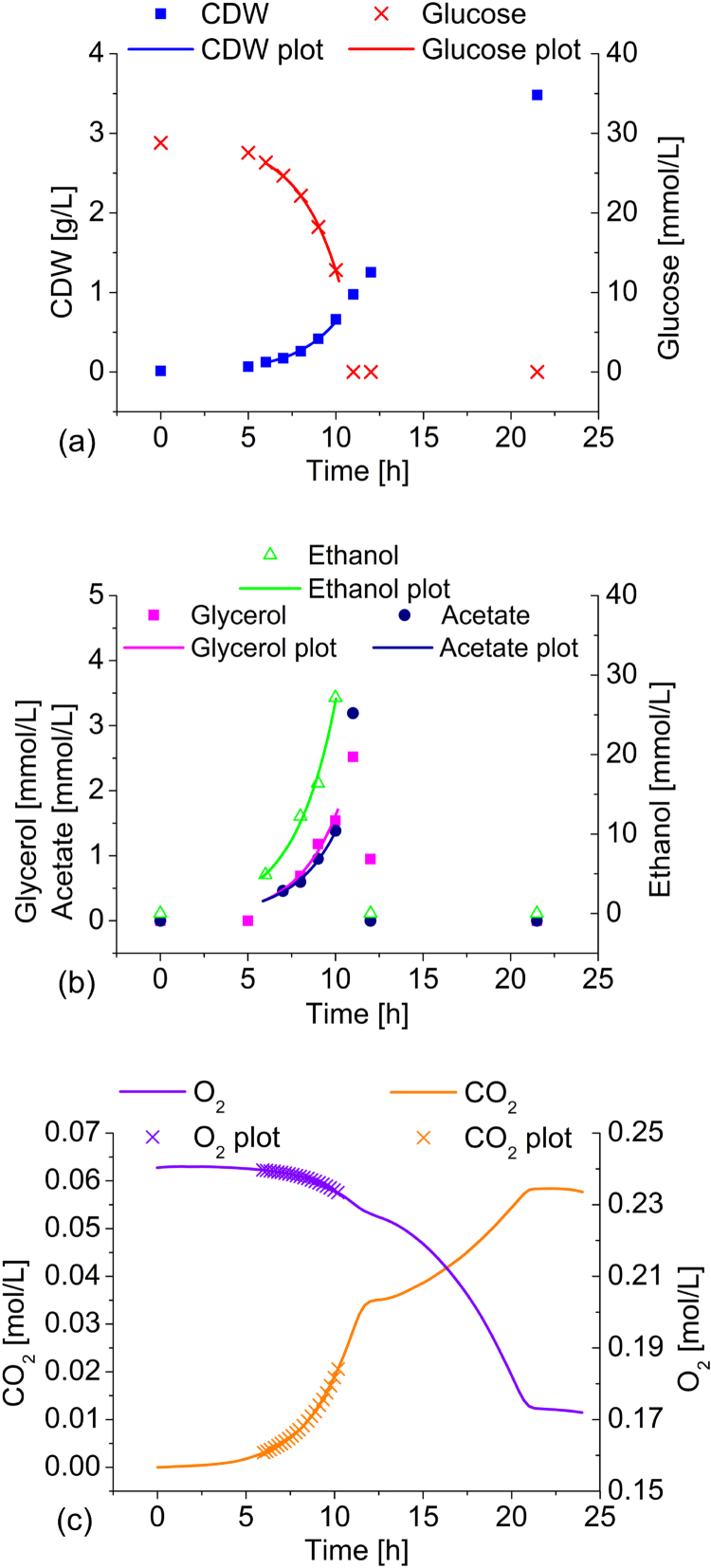
Physiology of *S. cerevisiae* CEN.PK 113-7D in minimal medium batch culture. A closed 1.3 L shake flask with a liquid volume of 50 mL Verduyn medium containing 5 g/L glucose was used at 30 °C. CDW, glucose, ethanol, acetate, and glycerol concentrations are indicated with symbols, while fitted values are represented by corresponding lines. The measured O_2_ and CO_2_ values are represented as lines because of the continuous nature of the measurements, and fitted data are represented with symbols.

**Fig. 2:**
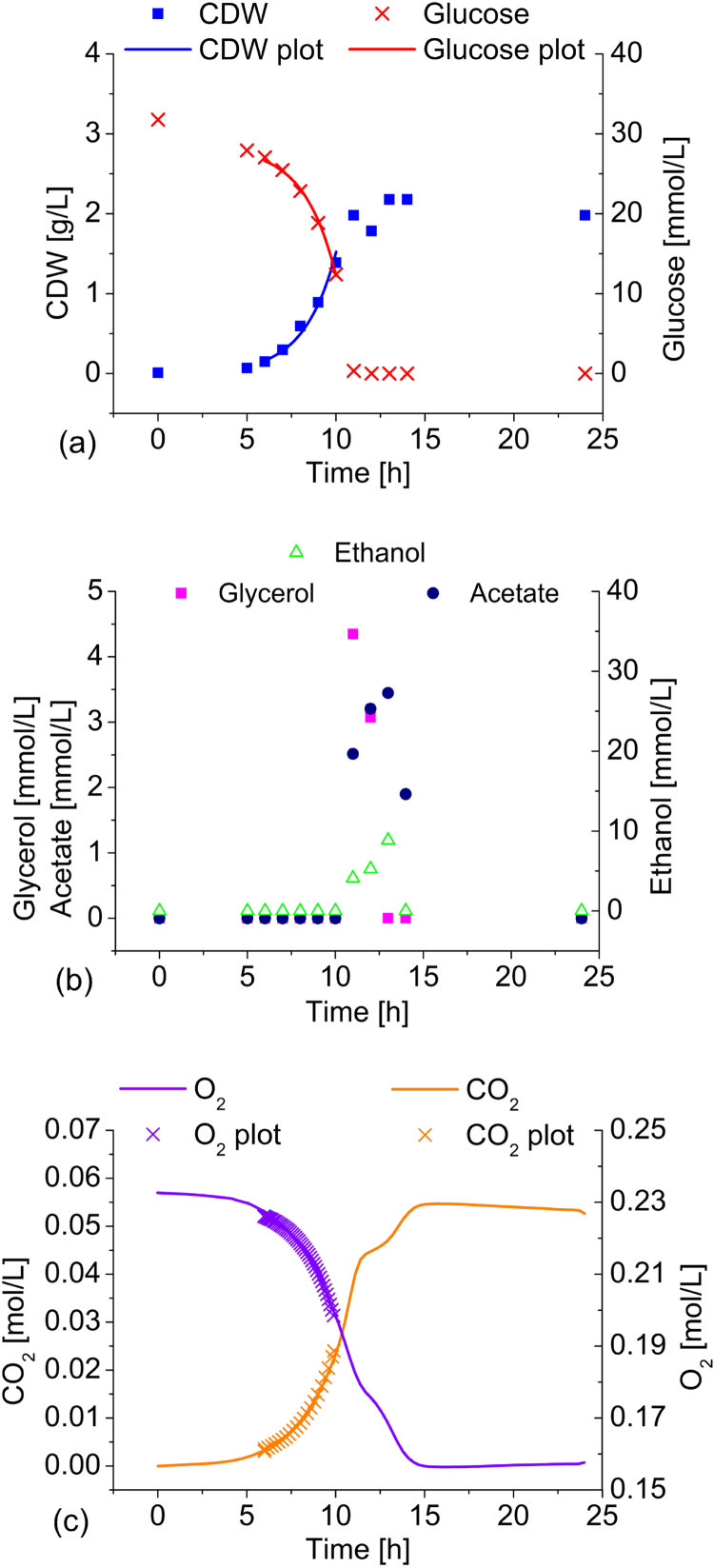
Physiology of *H. polymorpha* CLIB 421 in minimal medium batch culture. A closed 1.3 L shake flask with a liquid volume of 50 mL of Verduyn medium containing 5 g/L glucose was used at 40 °C. CDW, glucose, ethanol, acetate, and glycerol concentrations are indicated with symbols, while fitted values are represented by corresponding lines. The measured O_2_ and CO_2_ values are represented with lines because of the continuous nature of the measurements and fitted data are represented with symbols.

^13^C-MFA is applicable to cultures grown in a (pseudo-)metabolic and isotopic steady state. This was ensured here by growing *S. cerevisiae* and *H. polymorpha* in glucose minimal media in batch shake flask experiments and sampling for proteinogenic amino acid labeling analysis during the mid-exponential phase, approximately five generations after inoculation. Physiological data were monitored throughout the entire growth period (Figs. 1 and 2) to ensure well-founded selection of data exclusively derived from the exponential phase during growth on glucose for the calculation of uptake and production rates as well as OD_600_ to CDW correlations. The overlay with simulated data for the chosen exponential phases in the graphs indicate the quality of reaction rate estimates. As the accuracy of extracellular uptake and secretion rates considerably impacts the quality of the computed flux distribution (Nissen et al., 1997), the quality of acquired physiological data was revisited by determining the carbon balance (Table 3).

**Table 3:** Summary of experimentally acquired physiological data for *S. cerevisiae* at 30 °C and *H. polymorpha* at 40 °C. Both experiments were performed in 1.3 L shake flasks with 50 mL of Verduyn medium complemented with 5 g/L glucose.

	***S. cerevisiae***	***H. polymorpha***
**µ [h**^**−1**^**]**	0.4 ± 0.0	0.6 ± 0.0
**r**_**glucose**_	10.9 ± 1.12	5.9 ± 0.7
**[mmol g**_**CDW**_^**−1**^**h**^**−1**^**]**		
**r**_**glycerol**_	1.1 ± 0.1	0
**[mmol g**_**CDW**_^**−1**^**h**^**−1**^**]**		
**r**_**ethanol**_	17.6 ± 1.4	0
**[mmol g**_**CDW**_^**−1**^**h**^**−1**^**]**		
**r**_**acetate**_	0.9 ± 0.1	0
**[mmol g**_**CDW**_^**−1**^**h**^**−1**^**]**		
**r**_**O2**_	4.6 ± 0.0	12.1 ± 0.7
**[mmol g**_**CDW**_^**−1**^**h**^**−1**^**]**		
**measured r**_**CO2**_	12.8 ± 0.5	9.0 ± 0.5
**[mmol g**_**CDW**_^**−1**^**h**^**−1**^**]**		
**corrected r**_**CO2**_	15.9	12.6
**[mmol g**_**CDW**_^**−1**^**h**^**−1**^**]**		
**carbon recovery [%]**	109.5	94.8

The experimental data for *H. polymorpha* showed an accumulation of glycerol, acetate, and ethanol only after the considered exponential growth phase on glucose (Fig. 2), which is why the corresponding production rates were irrelevant for the flux analysis and are not represented with simulated data. The absence of the accumulation of byproducts is the central characteristic of *H. polymorpha*, corresponding to a higher growth rate. Growth rate and absent byproduct formation also result in significantly lower specific rates of glucose uptake and CO_2_ formation and a much higher rate of oxygen uptake.

### 3.4 Impact of constraints regarding amino acid biosynthesis localization on accuracy and resolvability of the flux distribution in *S. cerevisiae*

**Fig. 3:**
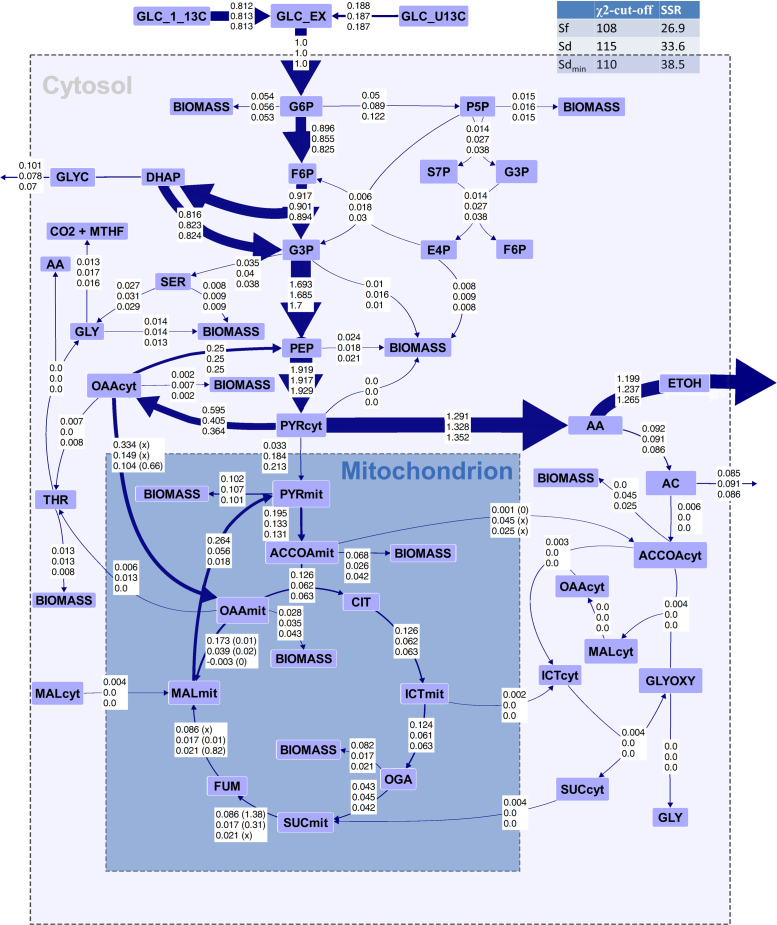
Simulated flux distributions of *S. cerevisiae*. The numbers in the boxes for each flux indicate the flux values of Sf in the upper line, the flux values of Sd in the middle line, and the flux values of Sd_min_ in the bottom line. All flux values were normalized to the glucose uptake rate. Reversible reactions are shown as net fluxes, with exchange reactions of the relevant fluxes shown in parentheses. The arrow thickness is scaled to Sf flux values respective to the arrow size of the glucose uptake flux. For relevant reversible reactions, the flux values of the exchange reaction are shown in parentheses, with x indicating unresolvable fluxes.

Intracellular flux distributions were computed by applying the calculated extracellular rates and amino acid labeling data as constraints and using the metabolic model either with the specified localization of amino acid biosynthesis (Sd) or without these additional constraints (Sf). Additionally, one solution was generated using a minimal set of localization constraints (Sd_min_); specifically, the acetolactate synthase involved in the biosynthesis of valine and leucine was defined as uniquely mitochondrial because of the unambiguous enzyme localization prediction results. The acquired physiological data (Table 3) and simulated flux distributions of *S. cerevisiae* (Fig. 3) corresponded well to previously published data with low TCA cycle and PPP activity of ca. 10%, high glycolytic flux, no activity of the glyoxylate shunt, and reductive activity of the malate dehydrogenase (Christen and Sauer, 2011, Wasylenko and Stephanopoulos, 2015), validating the experimental data as well as the applied computational methods.

The largest differences between the solutions Sf and Sd were found in the fluxes around the mitochondrial pyruvate and cytosolic oxaloacetate nodes as well as through the TCA cycle. Both flux solutions Sf and Sd displayed a reductive activity of the malate dehydrogenase, which indicates the high level of glucose repression on the TCA cycle. While the net and exchange reactions of acetyl-CoA transport were very small or zero in Sf, a net efflux of mitochondrial acetyl-CoA and an extensive exchange flux were computed for Sd. The flux through the phosphoenolpyruvate-pyruvate-oxaloacetate-phosphoenolpyruvate cycle was not resolvable and was therefore set to a PEP carboxykinase flux of 0.25 mol/mol_Glucose_ in the visualization, although simulated fluxes were very high in all three solutions. The origin of biomass precursors from the compartmental pools was not specified in the model, which resulted in the unlikely case that the vast majority of biomass was generated from mitochondrial precursors in all computed flux solutions. To avoid such likely artifacts, high-quality specifications of the biomass composition should be used when available. Here, it did not influence the relative distributions at branching points further downstream in the flux solutions. Notably, the differences in computed fluxes between Sd_min_, specifying only the localization of acetolactate synthase, and Sd were marginal compared with differences to Sf.

**Fig. 4:**
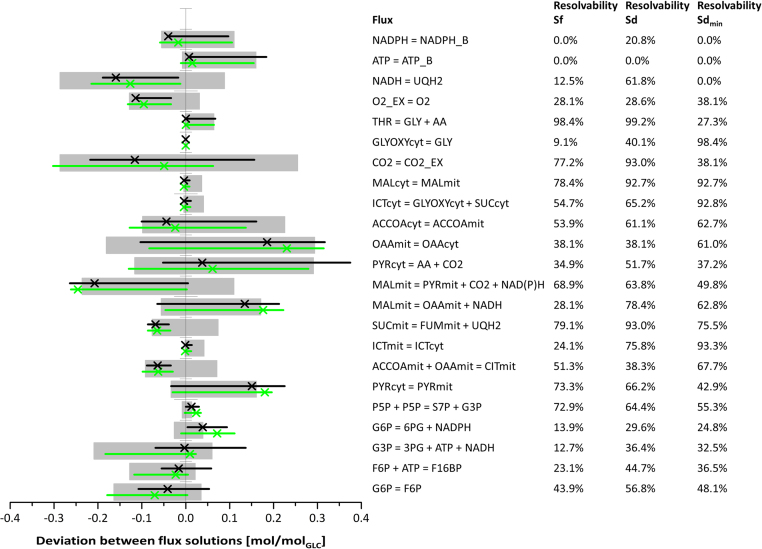
Excerpt of the sensitivity analyses for Sf, Sd, and Sd_min_ of the *S. cerevisiae* dataset comprising the major fluxes. Because absolute flux values differed substantially, the fluxes of solution Sf were normalized to the glucose uptake rate and were projected on the y-axis. The normalized fluxes of alternative flux solutions were scaled accordingly, i.e., plotted as the absolute deviation to the original Sf value. Black and green crosses represent the absolute deviations of the constrained solutions Sd and Sd_min_, respectively, from the unconstrained solution, Sf; gray bars and black and green lines represent the 95% confidence intervals of Sf, Sd, and Sd_min_, respectively. The reactions are specified by the reaction equations, shown on the right-hand side of the graph alongside the resolvability of each flux. The resolvability is defined as the ratio of the confidence interval and the stoichiometrically feasible flux range (Resolvability = (1 - confidence interval/stoichiometric range) x 100%) and indicates how well the flux parameter could be resolved from the ^13^C-labeling data.

To assess the significance of the divergent fluxes, flux confidence intervals were determined (Fig. 4). Remarkably, the confidence intervals of Sf for each flux included the flux values of Sd and, even for poorly resolved fluxes such as pyruvate decarboxylase flux, the solutions were highly similar. Overall, most of the Sf fluxes suffered a decrease in resolvability compared to Sd.

Furthermore, the solution based on a minimal set of compartmentation constraints, Sd_min_, showed a highly increased similarity to Sd compared to Sf and no significant increase of the flux intervals compared to Sd.

To further investigate the differences between the differently constrained models, the labeling patterns of compartmental pools of the amino acid precursors pyruvate, oxaloacetate, and acetyl-CoA were calculated by a forward isotopomer simulation in OpenFLUX (task 8) based on the computed flux distributions (Fig. 3). The results revealed that Sf differed from Sd only in its effect on mitochondrial acetyl-CoA and pyruvate labeling, while Sd and Sd_min_ were indistinguishable in the three MDVs. Furthermore, the deviations between mitochondrial and cytosolic labeling patterns of all metabolites in Sd were on the same scale or below the error of the mass distribution measurements of 0.005 Da. Accordingly, for the chosen ^13^C-labeling strategy and the flux distribution for respiro-fermentative growth of *S. cerevisiae* on glucose, none of the possible definitions of compartmental origin of amino acids would influence the labeling pattern of proteinogenic amino acids and, accordingly, the SSR. Thus, a flux solution based on any set of possible compartmentation constraints can be identical to Sd or at least within the confidence intervals of this solution. This is further confirmed by the fact that a forward simulation of amino acid labeling patterns based on the flux solution Sd with superimposed random compartmentation constraints resulted in no significant changes in the overall SSR (data not shown).

The differences between Sf and Sd were ascribed to a vast switch in the simulated compartmental origin of amino acids produced from pyruvate as a precursor (LEU, ILE, and VAL). With the unconstrained model, these amino acids were simulated to be synthesized, to a great extent, from cytosolic pyruvate, in contrast to the curated mitochondrial origin. This discrepancy can be attributed to the additional freedom of the unconstrained model, Sf, but does not result in deviations between flux solutions Sf and Sd beyond the confidence of the properly constrained solution, Sd.

**Fig. 5:**
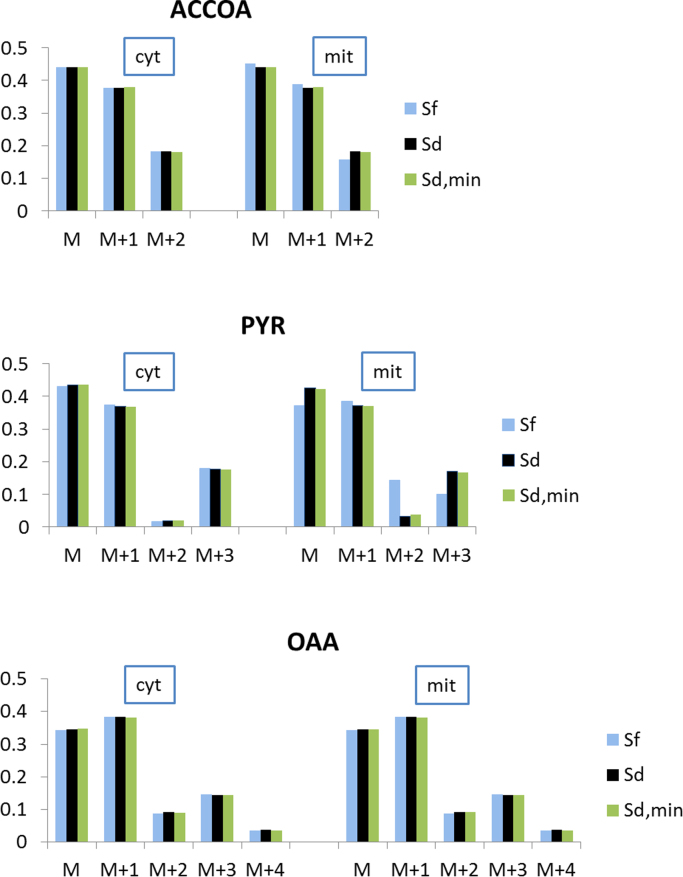
Simulated MDVs of OAA, ACCOA, and PYR based on the *S. cerevisiae* flux distributions Sf, Sd, and Sd_min_ shown in Fig. 3. M indicates the fraction of naturally labeled metabolites, and M+n indicates the fraction of metabolites with *n* labeled carbons.

The complete flux solutions for each model, the results of the simulated labeling of acetyl-CoA, pyruvate, and oxaloacetate, and the sensitivity analyses are included in the supplement (Fig. 5).

### 3.5 Impact of constraints regarding amino acid biosynthesis localization on accuracy and resolvability of the flux distribution in *H. polymorpha*

**Fig. 6:**
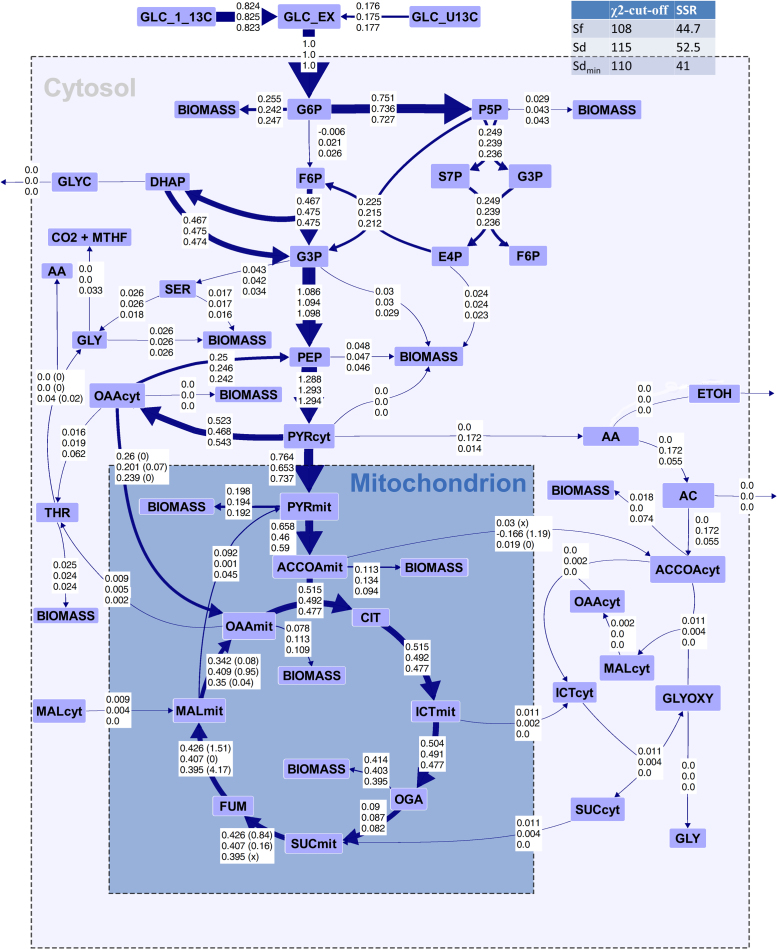
Simulated flux solution for *H. polymorpha.* Boxed numbers represent the flux values of Sf (upper value), Sd (middle value), and Sd_min_ (bottom value) for the respective reactions. The arrow thickness corresponds to Sf flux values. For relevant reversible reactions, the exchange flux is shown in parentheses, with x indicating unresolvable fluxes.

The flux solutions of *H. polymorpha* (Fig. 6) differed significantly from those of *S. cerevisiae.* The absence of the accumulation of byproducts resulted in high TCA cycle activity and higher fluxes feeding into biomass synthesis. Additionally, very high activity of the PPP of (ca. 75%) was simulated. A high PPP activity of ca. 50% was previously reported for this *H. polymorpha* strain during growth at 30 °C (Blank et al., 2005, Christen and Sauer, 2011). One possible explanation for the additional increase of PPP activity observed herein may be a higher NADPH demand for glutathione reductase-mediated protection against increased oxidative stress during aerobic growth at the elevated temperature of 40 °C (Grant, 2001, Sugiyama et al., 2000). High PPP activity may also be a general characteristic of this methylotrophic strain, as several PPP associated enzymes are involved in the pathway for methanol assimilation (Yurimoto et al., 2011).

**Fig. 7:**
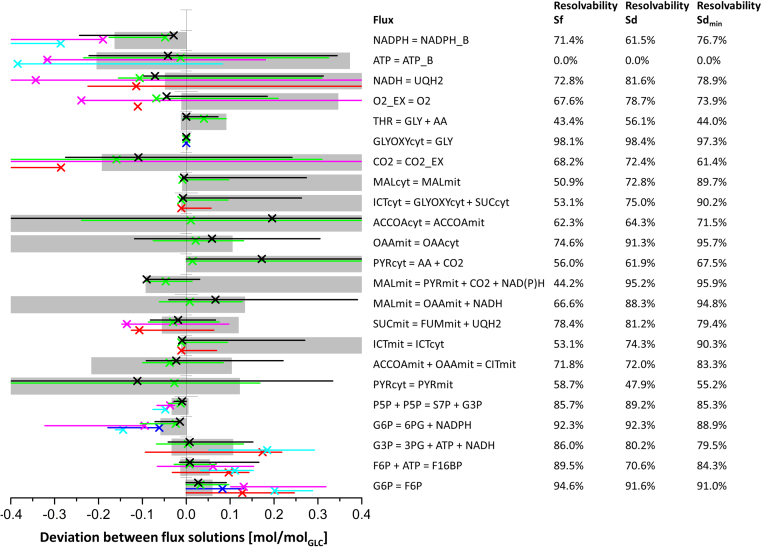
Excerpt of the sensitivity analyses of the *H. polymorpha* dataset comprising the major fluxes. A black x represents the absolute deviation of the constrained solution, Sd, from the undefined solution, Sf, thus assigning a value of zero to each respective flux of Sf. The gray bars display the 95% confidence intervals of Sf, and the black lines display the 95% confidence intervals of Sd. The resolvability of each flux is shown on the right-hand side. Results from flux solutions based on randomly assigned compartmentation constraints are shown for the selected fluxes in red (Sd_r1_), cyan (Sd_r2_), blue (Sd_r3_), and pink (Sd_r4_); Sd_min_ is shown in green. The lines indicating the confidence intervals are slightly shifted vertically to avoid a loss of information by overlapping of the lines.

As in the case of the *S. cerevisiae* dataset, the majority of differences between the *H. polymorpha* flux solutions Sf and Sd were found downstream of the pyruvate kinase reaction. In contrast to the unconstrained solution, Sf, the constrained solution, Sd, showed pyruvate decarboxylase activity and import of the cytosolic acetyl-CoA produced via this pathway into the mitochondria. With the free model, minimal activity of the malic enzyme and the glyoxylate shunt was computed, while no activity was simulated with the constrained model. The significance of all differences between Sf and Sd was evaluated by a sensitivity analysis of the fluxes, which revealed that the confidence intervals of all fluxes computed for Sf included the optimal solution of Sd, with the respiratory chain reactions being the sole exceptions (Fig. 7). There were no fluxes for which the confidence intervals of Sf and Sd did not broadly overlap. Additionally, similar to the *S. cerevisiae* flux solutions, the majority of biomass precursors were simulated to originate from the mitochondrial pools. This simulation result is questionable and is certainly a subject to be addressed for further improvement of the constraints, but it was not a determinant of flux ratios in the present study. In contrast to the *S. cerevisiae* dataset, the Sd_min_ solution of *H. polymorpha* was not explicitly more similar to Sd compared with Sf; rather, the Sd_min_ solution showed flux values that were often in between the values of Sf and Sd, which in some instances were closer to Sf and in others closer to Sd.

**Fig. 8:**
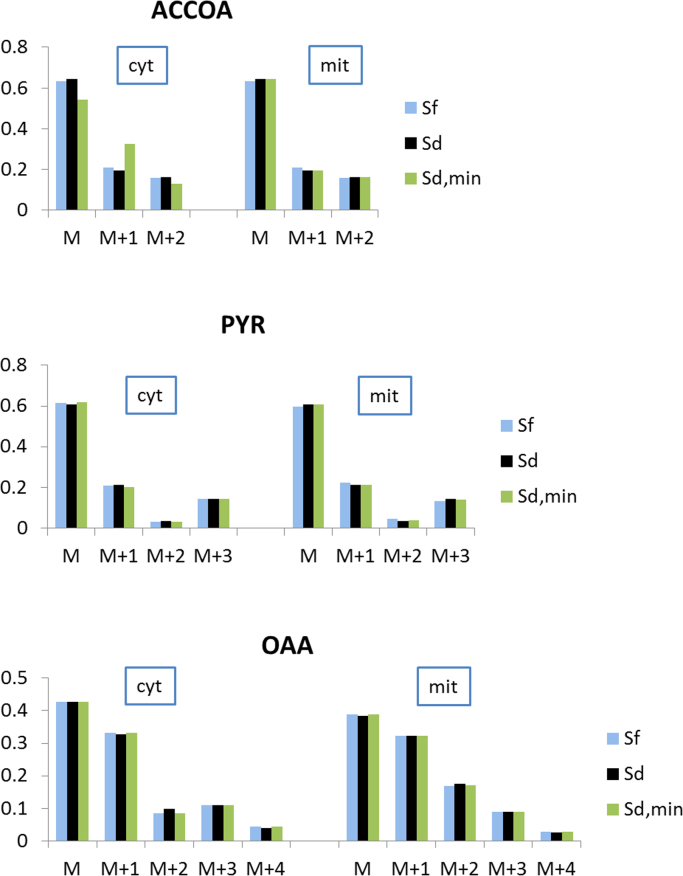
Simulated MDVs of OAA, ACCOA, and PYR based on the *H. polymorpha* flux distributions Sf, Sd, and Sd_min_ shown in Fig. 6.

The labeling patterns of acetyl-CoA, pyruvate, and oxaloacetate differed only marginally between the three solutions, with the exception of cytosolic acetyl-CoA computed for the solution Sd_min_. The absent exchange flux of acetyl-CoA between the mitochondria and cytosol prevented an equilibration of these two pools. However, as cytosolic acetyl-CoA was not used as an amino acid precursor in the flux solution, this difference had no further impact on the flux estimates. The simulated extensive exchange of acetyl-CoA across the mitochondrial membrane in Sd was reflected in equivalent labeling patterns of the respective parallel pools (Fig. 8). In contrast to *S. cerevisiae* flux solutions, the absent exchange of oxaloacetate resulted in significant differences in the compartmental labeling of oxaloacetate for all three solutions. The activity of the malic enzyme was reflected in a slightly different pyruvate labeling in Sf. The ratios of simulated compartmental origins of amino acids in Sf were reversed in comparison to Sd for all amino acids stemming from pyruvate; however, the rather poor resolvability of malic enzyme activity (Fig. 7), which is the sole determinant of pyruvate labeling, rendered these ratios unresolvable as well.

The differences between labeling patterns of mitochondrial and cytosolic acetyl-CoA, pyruvate, and, especially, oxaloacetate in all three solutions showed the potential for a higher influence of compartmentation constraints on the flux solutions compared with that seen in the *S. cerevisiae* dataset. To further strengthen this indication, four flux solutions were simulated, each with a different set of randomly assigned constraints regarding the compartmental origin of amino acids. Due to a lack of experimental data from a strain with definitive differences in the compartmentation of amino acid biosynthesis from the assumed compartmentation in *S. cerevisiae*, these simulations were meant to represent scenarios of improper use of compartmentation assumptions. These simulations resulted in a large number of fluxes outside of the flux confidence intervals of Sd or Sf (Fig. 7).

In contrast to the differences between Sf and Sd, the deviations of the flux values found in these additional solutions were often more significant. Most remarkably, there were fluxes with confidence intervals that did not overlap at all with those of Sf or Sd. Likewise, the SSRs of these additional solutions were significantly increased compared with those of Sf or Sd, with all but Sd_r3_ exceeding the χ^2^-cut-off values (Table 4). A solution with an SSR exceeding this cut-off value has to be disregarded, as this shows that the experimental error does not explain the differences between simulated and experimental labeling data and therefore points to errors in the metabolic model or an underestimation of the experimental error. Applying this criterion, most of the solutions derived from models with erroneous constraints would correctly be excluded, while solution Sd_r3_ would be rated as statistically acceptable, although it resulted in significant inaccuracy of the flux estimates.

**Table 4:** SSRs and χ^2^-cut-off values of flux solutions generated with randomly assigned constraints regarding compartmentation of amino acid biosynthesis.

	**Sd**_**r1**_	**Sd**_**r2**_	**Sd**_**r3**_	**Sd**_**r4**_
**χ**^**2**^**-cut-off**	119	119	120	120
**SSR**	223	159	72	172

These analyses indicate that improper use of constraining compartmentation assumptions can yield inaccurate results that may not always be revealed as false solutions by statistical measures.

The complete flux solutions for each model, the results of simulated labeling of acetyl-CoA, pyruvate, and oxaloacetate, and the sensitivity analyses are included in the supplement.

## 4 Conclusion

The presented analysis highlights the potential disadvantage of extrapolating established constraints, which were verified for cognate organisms or under different experimental conditions without reconfirmation for the application case. The comparison of flux solutions computed with and without compartmental localization constraints revealed that the retraction of these constraints did not significantly alter the simulated flux distributions, as the computed flux solutions showed extensive consistency. In contrast, an alarmingly adverse effect of randomly chosen constraints was shown, which represents the potentially equivalent negative consequence extrapolated curated or computationally predicted constraints would have when they are improperly assumed. These results do not allow a recommendation of total dismissal of compartmentation constraints but strongly support the demand for a more rigorous evaluation of such constraints and a rejection of constraints where there are no good reasons to extrapolate assumptions from other organisms or only vague computational indications.
