# Supplementary material for: A comprehensive evaluation of constraining amino acid biosynthesis in compartmented models for metabolic flux analysis
Source: Metab Eng Commun. 2017 Jul 11;5:34–44. doi: 10.1016/j.meteno.2017.07.001 (PMC5699530; doi:10.1016/j.meteno.2017.07.001)
Supplement: Supplementary file 2 — Supplementary material [file mmc2.docx]

*****

Highlighted in cyan are the resulting, most probable localizations from computational predictions with TargetP (T) and WoLF PSORT (WP)

*****

Valine/Leucine/Isoleucine

First step from pyruvate ILV6(regulatory)+ILV2(catalytic)

SC yeastgenome Regulatory subunit of acetolactate synthase

>ILV6 YCL009C SGDID:S000000515 mitochondrial (T+WP)

MLRSLLQSGHRRVVASSCATMVRCSSSSTSALAYKQMHRHATRPPLPTLDTPSWNANSAVSSIIYETPAPSRQPRKQHVLNCLVQNEPGVLSRVSGTLAARGFNIDSLVVCNTEVKDLSRMTIVLQGQDGVVEQARRQIEDLVPVYAVLDYTNSEIIKRELVMARISLLGTEYFEDLLLHHHTSTNAGAADSQELVAEIREKQFHPANLPASEVLRLKHEHLNDITNLTNNFGGRVVDISETSCIVELSAKPTRISAFLKLVEPFGVLECARSGMMALPRTPLKTSTEEAADEDEKISEIVDISQLPPG*

BLASTP Hit 1:

Score Expect Method Identities Positives Gaps

414 bits(1065) 9e-150 Compositional matrix adjust. 197/306(64%) 245/306(80%) 8/306(2%)

>jgi|Hanpo2|46543|e_gw1.2.493.1 mitochondrial (T+WP)

MISTKASRAIVGRVLRLARNNSSSTSALAYKNLHRNKKRPHLPTLDAPNWSAGAAVSSIIYETPLPSKAPKNQHVLNCLVQNEPGVLSLVSGTLAARGFNIDSLVVCNTEVKDLSRMTIVLDGQDAVIEQARRQIEDLVPVYAVLDYSNTEIIKRELLLARISLLGSEYFQELIAHHNEQVDEIPEDLHKSQFHPNNLPPSQALRQKYEHLDAITTLTKQFGGKVVDVSDRNCVVELCAKPSRVSAYIALVQPFGILEIARSGMMALPRTPLEAFSSEDGTDPSKDASDVVDASQLPPG*

Browsed regulatory acetolactate synthase; Identical to BLASTP Hit 1

>jgi|Hanpo2|15610|fgenesh1_kg.2_#_122_#_isotig04953

MISTKASRAIVGRVLRLARNNSSSTSALAYKNLHRNKKRPHLPTLDAPNWSAGAAVSSIIYETPLPSKAPKNQHVLNCLVQNEPGVLSLVSGTLAARGFNIDSLVVCNTEVKDLSRMTIVLDGQDAVIEQARRQIEDLVPVYAVLDYSNTEIIKRELLLARISLLGSEYFQELIAHHNEQVDEIPEDLHKSQFHPNNLPPSQALRQKYEHLDAITTLTKQFGGKVVDVSDRNCVVELCAKPSRVSAYIALVQPFGILEIARSGMMALPRTPLEAFSSEDGTDPSKDASDVVDASQLPPG*

SC yeastgenome Catalytic subunit of acetolactate synthase

>ILV2 YMR108W SGDID:S000004714 mitochondrial (T+WP)

MIRQSTLKNFAIKRCFQHIAYRNTPAMRSVALAQRFYSSSSRYYSASPLPASKRPEPAPSFNVDPLEQPAEPSKLAKKLRAEPDMDTSFVGLTGGQIFNEMMSRQNVDTVFGYPGGAILPVYDAIHNSDKFNFVLPKHEQGAGHMAEGYARASGKPGVVLVTSGPGATNVVTPMADAFADGIPMVVFTGQVPTSAIGTDAFQEADVVGISRSCTKWNVMVKSVEELPLRINEAFEIATSGRPGPVLVDLPKDVTAAILRNPIPTKTTLPSNALNQLTSRAQDEFVMQSINKAADLINLAKKPVLYVGAGILNHADGPRLLKELSDRAQIPVTTTLQGLGSFDQEDPKSLDMLGMHGCATANLAVQNADLIIAVGARFDDRVTGNISKFAPEARRAAAEGRGGIIHFEVSPKNINKVVQTQIAVEGDATTNLGKMMSKIFPVKERSEWFAQINKWKKEYPYAYMEETPGSKIKPQTVIKKLSKVANDTGRHVIVTTGVGQHQMWAAQHWTWRNPHTFITSGGLGTMGYGLPAAIGAQVAKPESLVIDIDGDASFNMTLTELSSAVQAGTPVKILILNNEEQGMVTQWQSLFYEHRYSHTHQLNPDFIKLAEAMGLKGLRVKKQEELDAKLEFVSTKGPVLLEVEVDKKVPVLPMVAGGSGLDEFINFDPEVERQQTELRHKRTGGKH*

BLASTP Hit 1:

Score Expect Method Identities Positives Gaps

997 bits(2578) 0.0 Compositional matrix adjust. 486/676(72%) 552/676(81%) 22/676(3%)

>jgi|Hanpo2|62281|estExt_Genewise1Plus.C_1_t30252 mitochondrial (T+WP)

MLRTSCKNTPAMRMVALRGARTVRAVRCKSSSASVFVDSRPTPAPSFNTSDRKGPSPLSSAPQMDSSFIGLTGGQIFHEMMQRHNVDTIFGYPGGAILPVYDAIYNSDKFNFVLPRHEQGAGHMAEGYARAVGKPGVVLVTSGPGATNVITPMADALADGVPMVVFTGQVPTSAIGTDAFQEADVVGISRSCTKWNVMVKNVAELPRRINEAFEIATSGRPGPVLVDLPKDVTAAILKEAIPVSSTLPSNTLNKITHAAATEFTTQCIQRAAALVNKAKKPILYVGAGILNSENGPKRLKELADKAQIPVTTTIQALGAFDQEDEKSLDMLGMHGSAVANSAMQNADLIIALGARFDDRVTGNVAKFAPEARLAAQENRGGIIHFEISPKNINKVVEATEAIEGDVTENLELFNQLVHPVASRPEWFDQIRAWKEKYPYAYQMETPGSKIKPQTLMREISKQANATGRDIVVTTGVGQHQMWAAQHFTWRKPRSFITSGGLGTMGFGLPAAIGAQIAKPDALVIDIDGDASFNMTLTELSSAVQANAPVKIVVLNNEEQGMVTQWQSLFYDYRYAHTHQSNPDFVKLGEAMGVKSFRVEDQSEMVDGVKKMIEYNDGPILMEVIVEKKVSVLPMVPGGCALDEFIVFDPEVEAQRDELRKQRTGGKH*

Browsed acetolactate synthase; Identical to BLASTP Hit 1 (-AS1..AS11)

>jgi|Hanpo2|15448|fgenesh1_kg.1_#_421_#_isotig01424

MRMVALRGARTVRAVRCKSSSASVFVDSRPTPAPSFNTSDRKGPSPLSSAPQMDSSFIGLTGGQIFHEMMQRHNVDTIFGYPGGAILPVYDAIYNSDKFNFVLPRHEQGAGHMAEGYARAVGKPGVVLVTSGPGATNVITPMADALADGVPMVVFTGQVPTSAIGTDAFQEADVVGISRSCTKWNVMVKNVAELPRRINEAFEIATSGRPGPVLVDLPKDVTAAILKEAIPVSSTLPSNTLNKITHAAATEFTTQCIQRAAALVNKAKKPILYVGAGILNSENGPKRLKELADKAQIPVTTTIQALGAFDQEDEKSLDMLGMHGSAVANSAMQNADLIIALGARFDDRVTGNVAKFAPEARLAAQENRGGIIHFEISPKNINKVVEATEAIEGDVTENLELFNQLVHPVASRPEWFDQIRAWKEKYPYAYQMETPGSKIKPQTLMREISKQANATGRDIVVTTGVGQHQMWAAQHFTWRKPRSFITSGGLGTMGFGLPAAIGAQIAKPDALVIDIDGDASFNMTLTELSSAVQANAPVKIVVLNNEEQGMVTQWQSLFYDYRYAHTHQSNPDFVKLGEAMGVKSFRVEDQSEMVDGVKKMIEYNDGPILMEVIVEKKVSVLPMVPGGCALDEFIVFDPEVEAQRDELRKQRTGGKH*

Isoleucine

First step from threonine

SC yeastgenome Threonine deaminnase

>ILV1 YER086W SGDID:S000000888 mitochondrial (T) unclear/cytosolic (WP)

MSATLLKQPLCTVVRQGKQSKVSGLNLLRLKAHLHRQHLSPSLIKLHSELKLDELQTDNTPDYVRLVLRSSVYDVINESPISQGVGLSSRLNTNVILKREDLLPVFSFKLRGAYNMIAKLDDSQRNQGVIACSAGNHAQGVAFAAKHLKIPATIVMPVCTPSIKYQNVSRLGSQVVLYGNDFDEAKAECAKLAEERGLTNIPPFDHPYVIAGQGTVAMEILRQVRTANKIGAVFVPVGGGGLIAGIGAYLKRVAPHIKIIGVETYDAATLHNSLQRNQRTPLPVVGTFADGTSVRMIGEETFRVAQQVVDEVVLVNTDEICAAVKDIFEDTRSIVEPSGALSVAGMKKYISTVHPEIDHTKNTYVPILSGANMNFDRLRFVSERAVLGEGKEVFMLVTLPDVPGAFKKMQKIIHPRSVTEFSYRYNEHRHESSSEVPKAYIYTSFSVVDREKEIKQVMQQLNALGFEAVDISDNELAKSHGRYLVGGASKVPNERIISFEFPERPGALTRFLGGLSDSWNLTLFHYRNHGADIGKVLAGISVPPRENLTFQKFLEDLGYTYHDETDNTVYQKFLKY*

BLASTP Hit 1:

Score Expect Method Identities Positives Gaps

750 bits(1937) 0.0 Compositional matrix adjust. 360/549(66%) 445/549(81%) 8/549(1%)

>jgi|Hanpo2|63499|estExt_Genewise1Plus.C_2_t30065 mitochondrial (T) unclear/cytosolic (WP)

MRKAAQLRLANTVQRLYSSAADICRRFPDLRPADVLPNGRPDYVKMILTSRVYDVIDESPISNAVSLSQKTNSTVMLKREDLLPVFSFKLRGAYNMIAHLSDERKSKGVIACSAGNHAQGVAYSAKEMGIPATIVMPVATPSIKFKNVSRLGSRVVLFGNDFDSAKAECERLTIEQGLTNIPPFDHPYVIAGQGTIAMELLRQVKSNKLSAVFVAVGGGGLLSGITAYLKRLAPHVKIIGVETFDADALKRSLEKGERVTLDSVGGFADGTAVRIVGEETFAVLQEFGVDEVVRVTTDEICAAIKDVFEDTRSLMEPSGAMTVAGLKRYVELHPEIDHSTKTYVPILSGANMNFDRLRFVSERAVLGEGKEVFLTVQIPDKPGTFARLNHIIDPRNVTEFSYRISADSKETGLANIFTSFSVIDRNKELGKVMKDLEQAGFKAHDLTDNEMAKSHGRYLVGGKVHVPGERIVSFEFPDRPGALTRFLSSMEMNWNLTLFHYRNHGDDVGKVLAGICVPPEDNAKFDKFLQNLGYKYNEETDNLVFKTFLTR*

BLASTP Hit 2:

Score Expect Method Identities Positives Gaps

717 bits(1851) 0.0 Compositional matrix adjust. 348/549(63%) 433/549(78%) 24/549(4%)

>jgi|Hanpo2|33574|estExt_fgenesh1_pm.C_2_t10427 mitochondrial (T) unclear/cytosolic (WP)

MRKAAQLRLANTVQRLYSSAADICRRFPDLRPADVLPNGRPDYVKMILTSRVYDVIDESPISNAVSLSQKTNSTVMLKREDLLPVFSFKLRGAYNMIAHLSDERKSKGVIACSAGNHAQGVAYSAKEMGIPATIVMPVATPSIKFKNVSRLGSRVVLFGNDFDSAKAECERLTIEQGLTNIPPFDHPYVIAGQGTIAMELLRQVKSNKLSAVFVAVGGGGLLSGITAYLKRLAPHVKIIGVETFDADALKRSLEKGERVTLDSVGGFADGTAVRIVGEETFAVLQEFGVDEVVRVTTDEICAAIKDVFEDTRSLMEPSGAMTVAGLKRYVELHPEIDHSTKTYVPILSGANMNFDRLRFVSERAVLGEGKEVFLTVQIPDKPGTFARLNHIIDPRNVTEFSYRISADSKETGLANIFTSFSVIDRNKELGKVMKDLEQAGFKAHDLTDNEMAKSHVSFEFPDRPGALTRFLSSMEMNWNLTLFHYRNHGDDVGKVLAGICVPPEDNAKFDKFLQNLGYKYNEETDNLVFKTFLTR*

Browsed threonine ammonia lyase; Identical to BLASTP Hit 1

>jgi|Hanpo2|15860|fgenesh1_kg.2_#_372_#_isotig01666

MRKAAQLRLANTVQRLYSSAADICRRFPDLRPADVLPNGRPDYVKMILTSRVYDVIDESPISNAVSLSQKTNSTVMLKREDLLPVFSFKLRGAYNMIAHLSDERKSKGVIACSAGNHAQGVAYSAKEMGIPATIVMPVATPSIKFKNVSRLGSRVVLFGNDFDSAKAECERLTIEQGLTNIPPFDHPYVIAGQGTIAMELLRQVKSNKLSAVFVAVGGGGLLSGITAYLKRLAPHVKIIGVETFDADALKRSLEKGERVTLDSVGGFADGTAVRIVGEETFAVLQEFGVDEVVRVTTDEICAAIKDVFEDTRSLMEPSGAMTVAGLKRYVELHPEIDHSTKTYVPILSGANMNFDRLRFVSERAVLGEGKEVFLTVQIPDKPGTFARLNHIIDPRNVTEFSYRISADSKETGLANIFTSFSVIDRNKELGKVMKDLEQAGFKAHDL

Leucine

AcCoA addition step

SC yeastgenome Alpha-isopropylmalate synthase

>LEU4 YNL104C SGDID:S000005048 mitochondrial/cytosolic cytosolic (T+WP)

MVKESIIALAEHAASRASRVIPPVKLAYKNMLKDPSSKYKPFNAPKLSNRKWPDNRITRAPRWLSTDLRDGNQSLPDPMSVEQKKEYFHKLVNIGFKEIEVSFPSASQTDFDFTRYAVENAPDDVSIQCLVQSREHLIKRTVEALTGAKKATIHTYLATSDMFREIVFNMSREEAISKAVEATKLVRKLTKDDPSQQATRWSYEFSPECFSDTPGEFAVEICEAVKKAWEPTEENPIIFNLPATVEVASPNVYADQIEYFATHITEREKVCISTHCHNDRGCGVAATELGMLAGADRVEGCLFGNGERTGNVDLVTVAMNMYTQGVSPNLDFSDLTSVLDVVERCNKIPVSQRAPYGGDLVVCAFSGSHQDAIKKGFNLQNKKRAQGETQWRIPYLPLDPKDIGRDYEAVIRVNSQSGKGGAAWVILRSLGLDLPRNMQIEFSSAVQDHADSLGRELKSDEISKLFKEAYNYNDEQYQAISLVNYNVEKFGTERRVFTGQVKVGDQIVDIEGTGNGPISSLVDALSNLLNVRFAVANYTEHSLGSGSSTQAASYIHLSYRRNADNEKAYKWGVGVSEDVGDSSVRAIFATINNIIHSGDVSIPSLAEVEGKNAAASGSA*

BLASTP Hit 1:

Score Expect Method Identities Positives Gaps

825 bits(2130) 0.0 Compositional matrix adjust. 394/597(66%) 473/597(79%) 17/597(2%)

>jgi|Hanpo2|50909|e_gw1.6.102.1 mitochondrial (T) unclear/mitochondrial (WP)

MFKRTLVLLGKSAAKPYANMLRDPSIKYSRFRGVDLPDRTWPNKVITKAPLWLSTDLRDGNQSLPDPMSVEQKKEYFHKLIEIGFKEIEVAFPSASQTDFDFTRYAVENAPEDVAIQALVQSREHLIRRTVDSLKGAKRAIVHTYLATSDLFRDVVFGMSREEAIAKAVETAKLVRSLTKDDPALQDTKWSYQFSPECFSDTPTEFALEICEAVKEAWEPTVDNPIIFNLPATVEVSGPHIYADQVEYFSRNISEREKVIISLHCHNDRGCGVAATELGLLAGADRVEGCLFGNGERTGNVDLVTVALNMYTDGVSPELDFSDLESVIDVVEKGNKIPVHCRSPYGGSLVVSAFSGSHQDAIKKGFAKQAEREAKGDMRWMIPYLPLDPKDIGRNYEAVIRVNSQSGKGGAAWVIERSLGLDLPREMQINFSKIVQDSADSLGRELKSDEIISLLQTSYNIDNCSNSALVLKDYKLDKESEFVTHIKAQFVYNGKEVEVEGTGNGPISSFVNAVAQAVGRDIELQKYAEHAVGKGSNTKAATYVLLSVGEDSQWGIGIHESITRASLNSIIASVNNLISSKPKTLHLKRSTA*

BLASTP Hit 2:

Score Expect Method Identities Positives Gaps

729 bits(1883) 0.0 Compositional matrix adjust. 353/578(61%) 428/578(74%) 19/578(3%)

>jgi|Hanpo2|68492|estExt_Genewise1Plus.C_7_t10273 cytosolic (T+WP)

MLPNPSEKYKKFVPLKLPNRQWPDRTFEKPPRWLSTDLRDGNQSLPDPMSVEEKKIYFQKLLDIGFKEIEVAFPSASQIDFDFTRYCVENAPQDVSIQVLSPCRPELIKRTVESLSGAKKAIVHLYLATSDCFRDVVFGLSKEETLKLATECTKLVRSLTKDDPKQKGTVWSYEFSPETFSDSDPDYVLEVCESVKAAWGPSKENPIIFNLPATVEMSTPNVYADQIEYFCTHISNRDAVCVSLHPHNDRGCAVGAAELAQLAGADRVEGCLFGNGERTGNVDLVTLALNLYTQGIHPNLDFSDMKSVIDVVEKCNKIPVHARAPYGGQLVVCAFSGSHQDAIKKGFERRKDNKGIWQIPYLPLDPQDIGRTYEAIIRVNSQSGKGGASWIILRNMELDLPRGLQISFSKVVQDATEKKGRELKANEIMDLFKNEYLLYNYEDGENLRPAGNLFKLDNYYLGNKDNKTRQLVVDMSNYDGTISYKVLGEGNGPISAFVNGLNTQFKSKFQVINYHEHSLGSDSNSKAAAYINCKVDDRHLIWGVGINEDVSTASFNSLLSVINQAIRKGVIKEMNV*

Browsed Alpha-isopropylmalate synthase; Identical to BLASTP Hit 1

Score Expect Method Identities Positives Gaps

825 bits(2130) 0.0 Compositional matrix adjust. 394/597(66%) 473/597(79%) 17/597(2%)

>jgi|Hanpo2|17174|fgenesh1_kg.6_#_135_#_isotig01068

MFKRTLVLLGKSAAKPYANMLRDPSIKYSRFRGVDLPDRTWPNKVITKAPLWLSTDLRDGNQSLPDPMSVEQKKEYFHKLIEIGFKEIEVAFPSASQTDFDFTRYAVENAPEDVAIQALVQSREHLIRRTVDSLKGAKRAIVHTYLATSDLFRDVVFGMSREEAIAKAVETAKLVRSLTKDDPALQDTKWSYQFSPECFSDTPTEFALEICEAVKEAWEPTVDNPIIFNLPATVEVSGPHIYADQVEYFSRNISEREKVIISLHCHNDRGCGVAATELGLLAGADRVEGCLFGNGERTGNVDLVTVALNMYTDGVSPELDFSDLESVIDVVEKGNKIPVHCRSPYGGSLVVSAFSGSHQDAIKKGFAKQAEREAKGDMRWMIPYLPLDPKDIGRNYEAVIRVNSQSGKGGAAWVIERSLGLDLPREMQINFSKIVQDSADSLGRELKSDEIISLLQTSYNIDNCSNSALVLKDYKLDKESEFVTHIKAQFVYNGKEVEVEGTGNGPISSFVNAVAQAVGRDIELQKYAEHAVGKGSNTKAATYVLLSVGEDSQWGIGIHESITRASLNSIIASVNNLISSKPKTLHLKRSTA*

SC yeastgenome Alpha-isopropylmalate synthase minor isozyme

>LEU9 YOR108W SGDID:S000005634 mitochondrial mitochondrial (T) cytosolic (WP)

MVKHSFIALAEHASKLRRSIPPVKLTYKNMLRDPSVKYRAFAPPKMVKRIWPDKTIQKAPRWLSTDLRDGNQSLPDPMSVAQKKEYFHKLINIGFKEIEVSFPSASQTDFDFTRYAVENAPDDVGIQCLVQSREHLIKRTVEALTGAKRATIHTYLATSDMFREIVFNMSREEAISKAVEATKLVRKLTKDDPSQQATRWSYEFSPECFSDTPGEFAVEICEAVKKAWEPTEENPIIFNLPATVEVASPNVYADQIEYFSTHITEREKVCISTHCHNDRGCGVAATELGMLAGADRVEGCLFGNGERTGNVDLVTVAMNMYTQGVSPNLDFSDLTSISEIVHRCNKIPIPPRAPYGGELVVSAFSGSHQDAIKKGFAIQNKKQAQGETRWRIPYLPLDPKDIGRDYEAVIRVNSQSGKGGAAWVIMRSLGLDVPRPMQVDFSNTLQKNADALGRELKSEEITKLFKETYNYNNNEHIYVTLLNYEVKKLNPERRALVGQVEINDKVVNIEGYGNGPISSLVDALSNLLNVKLSVQNYSEHSLGSGSATQAASFINLSYIKDINNHATSNMWGVGVSEDTGDASIKAVFATVNNIIHSGDVLLAE*

BLASTP Hit 1.

Score Expect Method Identities Positives Gaps

824 bits(2129) 0.0 Compositional matrix adjust. 392/597(66%) 475/597(79%) 17/597(2%)

>jgi|Hanpo2|59512|estExt_Genewise1.C_6_t10488 mitochondrial (T) unclear/mitochondrial (WP)

MFKRTLVLLGKSAAKPYANMLRDPSIKYSRFRGVDLPDRTWPNKVITKAPLWLSTDLRDGNQSLPDPMSVEQKKEYFHKLIEIGFKEIEVAFPSASQTDFDFTRYAVENAPEDVAIQALVQSREHLIRRTVDSLKGAKRAIVHTYLATSDLFRDVVFGMSREEAIAKAVETAKLVRSLTKDDPALQDTKWSYQFSPECFSDTPTEFALEICEAVKEAWEPTVDNPIIFNLPATVEVSGPHIYADQVEYFSRNISEREKVIISLHCHNDRGCGVAATELGLLAGADRVEGCLFGNGERTGNVDLVTVALNMYTDGVSPELDFSDLESVIDVVEKGNKIPVHCRSPYGGSLVVSAFSGSHQDAIKKGFAKQAEREAKGDMRWMIPYLPLDPKDIGRNYEAVIRVNSQSGKGGAAWVIERSLGLDLPREMQINFSKIVQDSADSLGRELKSDEIISLLQTSYNIDNCSNSALVLKDYKLDKESEFVTHIKAQFVYNGKEVEVEGTGNGPISSFVNAVAQAVGRDIELQKYAEHAVGKGSNTKAATYVLLSVGEDSQWGIGIHESITRASLNSIIASVNNLISSKPKTLHLKRSTA*

BLASTP Hit 2:

Score Expect Method Identities Positives Gaps

708 bits(1827) 0.0 Compositional matrix adjust. 343/579(59%) 429/579(74%) 20/579(3%)

>jgi|Hanpo2|68493|estExt_Genewise1Plus.C_7_t10274 cytosolic (T+WP)

MLPNPSEKYKKFVPLKLPNRQWPDRTFEKPPRWLSTDLRDGNQSLPDPMSVEEKKIYFQKLLDIGFKEIEVAFPSASQIDFDFTRYCVENAPQDVSIQVLSPCRPELIKRTVESLSGAKKAIVHLYLATSDCFRDVVFGLSKEETLKLATECTKLVRSLTKDDPKQKGTVWSYEFSPETFSDSDPDYVLEVCESVKAAWGPSKENPIIFNLPATVEMSTPNVYADQIEYFCTHISNRDAVCVSLHPHNDRGCAVGAAELAQLAGADRVEGCLFGNGERTGNVDLVTLALNLYTQGIHPNLDFSDMKSVIDVVEKCNKIPVHARAPYGGQLVVCAFSGSHQDAIKKGFERRKDNKGIWQIPYLPLDPQDIGRTYEAIIRVNSQSGKGGASWIILRNMELDLPRGLQISFSKVVQDATEKKGRELKANEIMDLFKNEYLLYNYEDGENLRPAGNLFKLDNYYLGNKDNKTRQLVVDMSNYDGTISYKVLGEGNGPISAFVNGLNTQFKSKFQVINYHEHSLGSDSNSKAAAYINCKVDDRHLIWGVGINEDVSTASFNSLLSVINQAIRKGVIKEMNV*

Browsed Alpha-isopropylmalate synthase; Identical to BLASTP Hit 2

>jgi|Hanpo2|95776|estExt_Genemark12.C_7_t10161

MLPNPSEKYKKFVPLKLPNRQWPDRTFEKPPRWLSTDLRDGNQSLPDPMSVEEKKIYFQKLLDIGFKEIEVAFPSASQIDFDFTRYCVENAPQDVSIQVLSPCRPELIKRTVESLSGAKKAIVHLYLATSDCFRDVVFGLSKEETLKLATECTKLVRSLTKDDPKQKGTVWSYEFSPETFSDSDPDYVLEVCESVKAAWGPSKENPIIFNLPATVEMSTPNVYADQIEYFCTHISNRDAVCVSLHPHNDRGCAVGAAELAQLAGADRVEGCLFGNGERTGNVDLVTLALNLYTQGIHPNLDFSDMKSVIDVVEKCNKIPVHARAPYGGQLVVCAFSGSHQDAIKKGFERRKDNKGIWQIPYLPLDPQDIGRTYEAIIRVNSQSGKGGASWIILRNMELDLPRGLQISFSKVVQDATEKKGRELKANEIMDLFKNEYLLYNYEDGENLRPAGNLFKLDNYYLGNKDNKTRQLVVDMSNYDGTISYKVLGEGNGPISAFVNGLNTQFKSKFQVINYHEHSLGSDSNSKAAAYINCKVDDRHLIWGVGINEDVSTASFNSLLSVINQAIRKGVIKEMNTYDDLDRDELLQRLVSLEKEFNAYIGESKELEEFLEGEVERLTKQAQTLEEANKRLQDQLDAASAQNIELTRQIGVSDDEKRAHVSQLNEKVRALEKRLIDTEVLNDGLESRLRILESSRDDEDARLGELIERLALLDSDNLGKDQLIGQLQLQIQALQRENKNLAQQCDKYEKFSKLMTINRTELKVAKFQTPPINE*

Aspartate/Threonine/Methionine

First step from Oxaloacetate

SC yeastgenome Mitochondrial aspartate aminotransferase

>AAT1 YKL106W SGDID:S000001589 mitochondrial (T+WP)

MLRTRLTNCSLWRPYYTSSLSRVPRAPPDKVLGLSEHFKKVKNVNKIDLTVGIYKDGWGKVTTFPSVAKAQKLIESHLELNKNLSYLPITGSKEFQENVMKFLFKESCPQFGPFYLAHDRISFVQTLSGTGALAVAAKFLALFISRDIWIPDPSWANHKNIFQNNGFENIYRYSYYKDGQIDIDGWIEQLKTFAYNNQQENNKNPPCIILHACCHNPTGLDPTKEQWEKIIDTIYELKMVPIVDMAYQGLESGNLLKDAYLLRLCLNVNKYPNWSNGIFLCQSFAKNMGLYGERVGSLSVITPATANNGKFNPLQQKNSLQQNIDSQLKKIVRGMYSSPPGYGSRVVNVVLSDFKLKQQWFKDVDFMVQRLHHVRQEMFDRLGWPDLVNFAQQHGMFYYTRFSPKQVEILRNNYFVYLTGDGRLSLSGVNDSNVDYLCESLEAVSKMDKLA*

BLASTP Hit 1:

Score Expect Method Identities Positives Gaps

282 bits(722) 6e-94 Compositional matrix adjust. 170/439(39%) 245/439(55%) 43/439(9%)

>jgi|Hanpo2|23516|fgenesh1_pm.2_#_462 mitochondrial (T+WP)

MLRVTSSRATKGLQFSKVAFFVAKRQESAWASVQAAPADKILGLTVLYNNDTNPSKINLGVGAYRDNDGKPWILPSVKAAEQVLAKTETNKEYVPIVGSPKFNELIKKMLYSHDEAGKKLLEDGRVLTAQGISGTGSLRVLGEFVRTFYPKSNKVLVPNPTWANHVAILEKAGLTTGKYSYYDYKTNALDEAGLLNDLASAEPGTVILLHACCHNPTGVDPELEQWDKILDVVSQKQLLPILDMAYQGFRSGSPIDDLAILFKFNKAVVDGKLSNFLLSQSFAKNMGLYGERVGSLSIITAGPEETTRVKSQLEKVIRPLYSSPPSHGSKLVEIILSDDTIYQQWLEDVRVMSDRLVEMRKLLHDKLKNTYKNPLNWDHLLNQKGMFCYTGLKEDQVKKLIDKSVYLTSDGRISIAGIYPANVDNLAKAIHEVTTN*

Browsed aspartate transaminase v1; Identical to BLASTP Hit 1

>jgi|Hanpo2|15884|fgenesh1_kg.2_#_396_#_isotig01439

MLRVTSSRATKGLQFSKVAFFVAKRQESAWASVQAAPADKILGLTVLYNNDTNPSKINLGVGAYRDNDGKPWILPSVKAAEQVLAKTETNKEYVPIVGSPKFNELIKKMLYSHDEAGKKLLEDGRVLTAQGISGTGSLRVLGEFVRTFYPKSNKVLVPNPTWANHVAILEKAGLTTGKYSYYDYKTNALDEAGLLNDLASAEPGTVILLHACCHNPTGVDPELEQWDKILDVVSQKQLLPILDMAYQGFRSGSPIDDLAILFKFNKAVVDGKLSNFLLSQSFAKNMGLYGERVGSLSIITAGPEETTRVKSQLEKVIRPLYSSPPSHGSKLVEIILSDDTIYQQWLEDVRVMSDRLVEMRKLLHDKLKNTYKNPLNWDHLLNQKGMFCYTGLKEDQVKKLIDKSVYLTSDGRISIAGIYPANVDNLAKAIHEVTTN*

BLASTP Hit 2:

Score Expect Method Identities Positives Gaps

267 bits(683) 2e-88 Compositional matrix adjust. 154/440(35%) 242/440(55%) 43/440(9%)

>jgi|Hanpo2|28639|estExt_fgenesh1_pg.C_1_t10341 cytosolic (T+WP)

MTRSFSIENIPQLPPDPLFGLKARYSEDPRPNKVDLGIGAYRDNDGKPWILPSVRLAENLLQNSKEYNHEYLAISGYKAFTDAAARIILGKQSKAIAEDRLVSIQTLSGTGALHVAGKFLKEFYVSNTSKEPPTVYLSKPTWANHEQIFGYLGLKTASYPYWNNETKTLDLDGFVRSIEQAPEGSVFLLHATAHNPTGLDPKPDQWLKILQAIEKGNHLALFDSAYQGFSSGSLDKDAWAVREAVDKKYSFPIVVCQSFAKNAGMYGERVGAVHVVLPEHDTALNKAVLSQLSKIIRSEISNPPAYGAKIVSLILNTPELMKQWEDDLVTMSSRISKMRQTLTAELEKLGTPGSWKHIVEQQGMFSFTGLTPAQVERLEKKHGVYLVSSGRASIAGLNEGNVKHVAKCIDEVVRSV*

Browsed aspartate transaminase v2; Identical to BLASTP Hit 2

>jgi|Hanpo2|96725|estExt_Genewise1Plus2.C_1_t20122

MTRSFSIENIPQLPPDPLFGLKARYSEDPRPNKVDLGIGAYRDNDGKPWILPSVRLAENLLQNSKEYNHEYLAISGYKAFTDAAARIILGKQSKAIAEDRLVSIQTLSGTGALHVAGKFLKEFYVSNTSKEPPTVYLSKPTWANHEQIFGYLGLKTASYPYWNNETKTLDLDGFVRSIEQAPEGSVFLLHATAHNPTGLDPKPDQWLKILQAIEKGNHLALFDSAYQGFSSGSLDKDAWAVREAVDKKYSFPIVVCQSFAKNAGMYGERVGAVHVVLPEHDTALNKAVLSQLSKIIRSEISNPPAYGAKIVSLILNTPELMKQWEDDLVTMSSRISKMRQTLTAELEKLGTPGSWKHIVEQQGMFSFTGLTPAQVERLEKKHGVYLVSSGRASIAGLNEGNVKHVAKCIDEVVRSV*

SC yeastgenome Cytosolic aspartate aminotransferase

>AAT2 YLR027C SGDID:S000004017 cytosolic (T+WP)

MSATLFNNIELLPPDALFGIKQRYGQDQRATKVDLGIGAYRDDNGKPWVLPSVKAAEKLIHNDSSYNHEYLGITGLPSLTSNAAKIIFGTQSDAFQEDRVISVQSLSGTGALHISAKFFSKFFPDKLVYLSKPTWANHMAIFENQGLKTATYPYWANETKSLDLNGFLNAIQKAPEGSIFVLHSCAHNPTGLDPTSEQWVQIVDAIASKNHIALFDTAYQGFATGDLDKDAYAVRLGVEKLSTVSPVFVCQSFAKNAGMYGERVGCFHLALTKQAQNKTIKPAVTSQLAKIIRSEVSNPPAYGAKIVAKLLETPELTEQWHKDMVTMSSRITKMRHALRDHLVKLGTPGNWDHIVNQCGMFSFTGLTPQMVKRLEETHAVYLVASGRASIAGLNQGNVEYVAKAIDEVVRFYTIEAKL*

BLASTP Hit 1: Identical to BLASTP Hit 2 von AAT1

>jgi|Hanpo2|53393|estExt_Genewise1.C_1_t20125 cytosolic (T+WP)

MTRSFSIENIPQLPPDPLFGLKARYSEDPRPNKVDLGIGAYRDNDGKPWILPSVRLAENLLQNSKEYNHEYLAISGYKAFTDAAARIILGKQSKAIAEDRLVSIQTLSGTGALHVAGKFLKEFYVSNTSKEPPTVYLSKPTWANHEQIFGYLGLKTASYPYWNNETKTLDLDGFVRSIEQAPEGSVFLLHATAHNPTGLDPKPDQWLKILQAIEKGNHLALFDSAYQGFSSGSLDKDAWAVREAVDKKYSFPIVVCQSFAKNAGMYGERVGAVHVVLPEHDTALNKAVLSQLSKIIRSEISNPPAYGAKIVSLILNTPELMKQWEDDLVTMSSRISKMRQTLTAELEKLGTPGSWKHIVEQQGMFSFTGLTPAQVERLEKKHGVYLVSSGRASIAGLNEGNVKHVAKCIDEVVRSV*

Browsed aspartate transaminase v2; Identical to BLASTP Hit 1

>jgi|Hanpo2|96725|estExt_Genewise1Plus2.C_1_t20122

MTRSFSIENIPQLPPDPLFGLKARYSEDPRPNKVDLGIGAYRDNDGKPWILPSVRLAENLLQNSKEYNHEYLAISGYKAFTDAAARIILGKQSKAIAEDRLVSIQTLSGTGALHVAGKFLKEFYVSNTSKEPPTVYLSKPTWANHEQIFGYLGLKTASYPYWNNETKTLDLDGFVRSIEQAPEGSVFLLHATAHNPTGLDPKPDQWLKILQAIEKGNHLALFDSAYQGFSSGSLDKDAWAVREAVDKKYSFPIVVCQSFAKNAGMYGERVGAVHVVLPEHDTALNKAVLSQLSKIIRSEISNPPAYGAKIVSLILNTPELMKQWEDDLVTMSSRISKMRQTLTAELEKLGTPGSWKHIVEQQGMFSFTGLTPAQVERLEKKHGVYLVSSGRASIAGLNEGNVKHVAKCIDEVVRSV*

BLASTP Hit 2: Identical to BLASTP Hit 1 von AAT1

>jgi|Hanpo2|7242|gm1.1500_g mitochondrial (T+WP)

MLRVTSSRATKGLQFSKVAFFVAKRQESAWASVQAAPADKILGLTVLYNNDTNPSKINLGVGAYRDNDGKPWILPSVKAAEQVLAKTETNKEYVPIVGSPKFNELIKKMLYSHDEAGKKLLEDGRVLTAQGISGTGSLRVLGEFVRTFYPKSNKVLVPNPTWANHVAILEKAGLTTGKYSYYDYKTNALDEAGLLNDLASAEPGTVILLHACCHNPTGVDPELEQWDKILDVVSQKQLLPILDMAYQGFRSGSPIDDLAILFKFNKAVVDGKLSNFLLSQSFAKNMGLYGERVGSLSIITAGPEETTRVKSQLEKVIRPLYSSPPSHGSKLVEIILSDDTIYQQWLEDVRVMSDRLVEMRKLLHDKLKNTYKNPLNWDHLLNQKGMFCYTGLKEDQVKKLIDKSVYLTSDGRISIAGIYPANVDNLAKAIHEVTTN*

Browsed aspartate transaminase v1; Identical to BLASTP Hit 2

>jgi|Hanpo2|15884|fgenesh1_kg.2_#_396_#_isotig01439

MLRVTSSRATKGLQFSKVAFFVAKRQESAWASVQAAPADKILGLTVLYNNDTNPSKINLGVGAYRDNDGKPWILPSVKAAEQVLAKTETNKEYVPIVGSPKFNELIKKMLYSHDEAGKKLLEDGRVLTAQGISGTGSLRVLGEFVRTFYPKSNKVLVPNPTWANHVAILEKAGLTTGKYSYYDYKTNALDEAGLLNDLASAEPGTVILLHACCHNPTGVDPELEQWDKILDVVSQKQLLPILDMAYQGFRSGSPIDDLAILFKFNKAVVDGKLSNFLLSQSFAKNMGLYGERVGSLSIITAGPEETTRVKSQLEKVIRPLYSSPPSHGSKLVEIILSDDTIYQQWLEDVRVMSDRLVEMRKLLHDKLKNTYKNPLNWDHLLNQKGMFCYTGLKEDQVKKLIDKSVYLTSDGRISIAGIYPANVDNLAKAIHEVTTN*

Threonine/Methionine

First step from Aspartat to homoserine

SC yeastgenome Cytosolic aspartate kinase

>HOM3 YER052C SGDID:S000000854 unclear (T) cytosolic (WP)

MPMDFQPTSSHSNWVVQKFGGTSVGKFPVQIVDDIVKHYSKPDGPNNNVAVVCSARSSYTKAEGTTSRLLKCCDLASQESEFQDIIEVIRQDHIDNADRFILNPALQAKLVDDTNKELELVKKYLNASKVLGEVSSRTVDLVMSCGEKLSCLFMTALCNDRGCKAKYVDLSHIVPSDFSASALDNSFYTFLVQALKEKLAPFVSAKERIVPVFTGFFGLVPTGLLNGVGRGYTDLCAALIAVAVNADELQVWKEVDGIFTADPRKVPEARLLDSVTPEEASELTYYGSEVIHPFTMEQVIRAKIPIRIKNVQNPLGNGTIIYPDNVAKKGESTPPHPPENLSSSFYEKRKRGATAITTKNDIFVINIHSNKKTLSHGFLAQIFTILDKYKLVVDLISTSEVHVSMALPIPDADSLKSLRQAEEKLRILGSVDITKKLSIVSLVGKHMKQYIGIAGTMFTTLAEEGINIEMISQGANEINISCVINESDSIKALQCIHAKLLSERTNTSNQFEHAIDERLEQLKRLGI*

NO BLAST HITS / NO BROWSE HITS

Glutamate/Proline

First step from Oxoglutarate

SC yeastgenome glutamate dehydrogenase

>GDH1 YOR375C SGDID:S000005902 cytosolic (T+WP)

MSEPEFQQAYEEVVSSLEDSTLFEQHPEYRKVLPIVSVPERIIQFRVTWENDKGEQEVAQGYRVQYNSAKGPYKGGLRFHPSVNLSILKFLGFEQIFKNSLTGLDMGGGKGGLCVDLKGRSNNEIRRICYAFMRELSRHIGQDTDVPAGDIGVGGREIGYLFGAYRSYKNSWEGVLTGKGLNWGGSLIRPEATGYGLVYYTQAMIDYATNGKESFEGKRVTISGSGNVAQYAALKVIELGGTVVSLSDSKGCIISETGITSEQVADISSAKVNFKSLEQIVNEYSTFSENKVQYIAGARPWTHVQKVDIALPCATQNEVSGEEAKALVAQGVKFIAEGSNMGSTPEAIAVFETARSTATGPSEAVWYGPPKAANLGGVAVSGLEMAQNSQRITWTSERVDQELKRIMINCFNECIDYAKKYTKDGKVLPSLVKGANIASFIKVSDAMFDQGDVF*

BLASTP Hit 1:

Score Expect Method Identities Positives Gaps

652 bits(1681) 0.0 Compositional matrix adjust. 319/452(71%) 374/452(82%) 2/452(0%)

>jgi|Hanpo2|6581|gm1.839_g cytosolic (T+WP)

MVQPQEPEFEQAFNELYSTLKESTLFQKHPEYEKVIPVVSVPERIIQFRVTWEDDQGNVQVNRGYRVQFNSALGPYKGGLRLHPSVNLSILKFLGYEQIFKNALTGLSMGGGKGGSDFDPKGKSDNEIRRFCTAFIRQLARHIGQFTDVPAGDIGTTGREIGFMFGAYKQMQNQFEGVLTGKGLTWGGSLIRPEATGYGLVYYVEKMIEKATNGKESFAGKRVAISGSGNVAQYAALKVIELGGTVVSLSDSKGCIICQSGITKEHIEAIIEAKVKFMSLKDIIGEYSAFETTPVQYIDGARPWVHAGKVDVALPCATQNEVSGEEAKALVAVGCKYLAEGSNMGSTQEAIDVFEAERKSASSVGDSIWYAPGKAANCGGVAVSGLEMAQNSQRVQWSAEEVDKKLKDIMVNCFNTCYETAVSYSAETNKDTLPSLVMGANVAGFVKVAEAMKAQGDWW*

Browsed glutamate dehydrogenase v1,

>jgi|Hanpo2|82333|estExt_fgenesh1_pm2.C_30427 cytosolic WP / unclear T

MTLEQKLGGLTIHNNFSATSFQNGPGYKSAVFSGKEEQMVQVMDELDSTGFIPENLIESETRYFYESLGIDDTYFASESVSVIVSHILALYSAKVDAFARGLTDRPFLHHRRETDDHAVYFDAAGWDDFEQEIDDKYLDRAVEETPYRLESFNSRLPTGDVVKCQFVYKCKFHTQGVSASSDIELVGDSTFLKTATPHTKLLYSEVLNELAATEGPVIKHFTVAATGEQRIIIGFRRNSSPRYSSALSTLAKYYNVNLRRKYVENFANGYSIISMYIDYTDGLSPDATIYQMAKEASLLYCIPNNMFYSKFASGEMSIQESIYAHCGVIFVTHFLNRLGPEFNALRELLSHSVSPQHAEIIDKIKKRLTSETYTQSYIAECFENNKDLISQLYRHFVDHHYTSTSLQSTLSYQRVEKVSPIENDEDFEKALNRSASANEGHKLVLRALYSFNKSVLKTNFFVTSKIALSFRLDPSFLPKTEYPQTPFGMFFVVGSDFRGFHIRFRDVARGGIRIVKSRSKDNYFTNMRTLFDENYNLASTQQRKNKDIPEGGSKGVILLNPGAAQERPKECFTKYIDSLLDLLIKDPRKETIVDLYNKPEMLYMGPDEYTAGFVDWATLHARKRGAKYGFPWKAFFTGKSPTLGGIPHDKYGMTSLSVRAYTEKIYEKLGITDLGKITKVQIAGGDGDLGSNEIKLSREEKYVAIVDGFGVVVDEDGLDKQELLRLAHERKGNQHFDKSKLGPKGYLVLVDDTDVKLPDGRVVASGLTFRNNFHLTLKENFPNNFIKLFVPCGGRPNSLDANNVHSLIDEKTGKSIIPFIVEGANLFITQPAKLMLEKAGAIVFKDASTNKGGVTSSSLEVLAALSFDDEGFLKHMCVDSATNKVPEFYMQYVKEVQQIVVRNARNEFELLWSLKEQTGKPFSILSDELSMAINKLADELASSKELWDDDEKFRNDVLTDALPHLLLKQIGIQTILKRVPTAYLRAIFATRLASEFVYSRGIDSNPAKFLEFISSLKRRHKKKI*

Browsed glutamate dehydrogenase v2, identical to BLASTP Hit 1

>jgi|Hanpo2|92383|estExt_Genemark12.C_2_t10021

MVQPQEPEFEQAFNELYSTLKESTLFQKHPEYEKVIPVVSVPERIIQFRVTWEDDQGNVQVNRGYRVQFNSALGPYKGGLRLHPSVNLSILKFLGYEQIFKNALTGLSMGGGKGGSDFDPKGKSDNEIRRFCTAFIRQLARHIGQFTDVPAGDIGTTGREIGFMFGAYKQMQNQFEGVLTGKGLTWGGSLIRPEATGYGLVYYVEKMIEKATNGKESFAGKRVAISGSGNVAQYAALKVIELGGTVVSLSDSKGCIICQSGITKEHIEAIIEAKVKFMSLKDIIGEYSAFETTPVQYIDGARPWVHAGKVDVALPCATQNEVSGEEAKALVAVGCKYLAEGSNMGSTQEAIDVFEAERKSASSVGDSIWYAPGKAANCGGVAVSGLEMAQNSQRVQWSAEEVDKKLKDIMVNCFNTCYETAVSYSAETNKDTLPSLVMGANVAGFVKVAEAMKAQGDWW*

>GDH3 YAL062W SGDID:S000000058 YG:mitochondrial cytosolic (T+WP)

MTSEPEFQQAYDEIVSSVEDSKIFEKFPQYKKVLPIVSVPERIIQFRVTWENDNGEQEVAQGYRVQFNSAKGPYKGGLRFHPSVNLSILKFLGFEQIFKNALTGLDMGGGKGGLCVDLKGKSDNEIRRICYAFMRELSRHIGKDTDVPAGDIGVGGREIGYLFGAYRSYKNSWEGVLTGKGLNWGGSLIRPEATGFGLVYYTQAMIDYATNGKESFEGKRVTISGSGNVAQYAALKVIELGGIVVSLSDSKGCIISETGITSEQIHDIASAKIRFKSLEEIVDEYSTFSESKMKYVAGARPWTHVSNVDIALPCATQNEVSGDEAKALVASGVKFVAEGANMGSTPEAISVFETARSTATNAKDAVWFGPPKAANLGGVAVSGLEMAQNSQKVTWTAERVDQELKKIMINCFNDCIQAAQEYSTEKNTNTLPSLVKGANIASFVMVADAMLDQGDVF*

BLASTP Hit 1; Identical to BLASTP Hit 1 GDH1:

Score Expect Method Identities Positives Gaps

657 bits(1694) 0.0 Compositional matrix adjust. 316/452(70%) 377/452(83%) 0/452(0%)

>jgi|Hanpo2|54224|estExt_Genewise1.C_2_t10034 cytosolic (T+WP)

MVQPQEPEFEQAFNELYSTLKESTLFQKHPEYEKVIPVVSVPERIIQFRVTWEDDQGNVQVNRGYRVQFNSALGPYKGGLRLHPSVNLSILKFLGYEQIFKNALTGLSMGGGKGGSDFDPKGKSDNEIRRFCTAFIRQLARHIGQFTDVPAGDIGTTGREIGFMFGAYKQMQNQFEGVLTGKGLTWGGSLIRPEATGYGLVYYVEKMIEKATNGKESFAGKRVAISGSGNVAQYAALKVIELGGTVVSLSDSKGCIICQSGITKEHIEAIIEAKVKFMSLKDIIGEYSAFETTPVQYIDGARPWVHAGKVDVALPCATQNEVSGEEAKALVAVGCKYLAEGSNMGSTQEAIDVFEAERKSASSVGDSIWYAPGKAANCGGVAVSGLEMAQNSQRVQWSAEEVDKKLKDIMVNCFNTCYETAVSYSAETNKDTLPSLVMGANVAGFVKVAEAMKAQGDWW*

Lysine

AcCoA addition step to mitochondrial OGA

SC yeast genome Homocitrate synthase

>LYS21 YDL131W SGDID:S000002289 YG:nucleus cytosolic (T+WP)

MSENNEFQSVTESTTAPTTSNPYGPNPADYLSNVKNFQLIDSTLREGEQFANAFFDTEKKIEIARALDDFGVDYIELTSPVASEQSRKDCEAICKLGLKAKILTHIRCHMDDARVAVETGVDGVDVVIGTSKFLRQYSHGKDMNYIAKSAVEVIEFVKSKGIEIRFSSEDSFRSDLVDLLNIYKTVDKIGVNRVGIADTVGCANPRQVYELIRTLKSVVSCDIECHFHNDTGCAIANAYTALEGGARLIDVSVLGIGERNGITPLGGLMARMIVAAPDYVRSKYKLHKIRDIENLVADAVEVNIPFNNPITGFCAFTHKAGIHAKAILANPSTYEILDPHDFGMKRYIHFANRLTGWNAIKSRVDQLNLNLTDDQIKEVTAKIKKLGDVRPLNIDDVDSIIKDFHAELSTPLLKPVNKGTDDDNIDISNGHVSKKAKVTK*

BLASTP Hit 1:

Score Expect Method Identities Positives Gaps

762 bits(1968) 0.0 Compositional matrix adjust. 361/414(87%) 391/414(94%) 0/414(0%)

>jgi|Hanpo2|67771|estExt_Genewise1Plus.C_6_t20016 cytosolic (T+WP)

MSSLDQYQKVSEEQHKKVQVNPYGPNPSDYLSNVRSFQLIESTLREGEQFANAFFSTEKKIEIAKALDDFGVDYIELTSPVASEQSRIDCEAICKLGLKAKILTHIRCHMDDAKVAVETGVDGVDVVIGTSQFLRQYSHGKDMSYITKSAVEVIEFVKSKGIEIRFSSEDSFRSDIVDLLNIYKTVDKIGVNRVGIADTVGCANPRQVYELVKTLKSVVSCDIECHFHNDTGCAIANAYTALEAGAKLIDVSVLGIGERNGITPLGGLMARMIAADREYVMSKYKLHKLRDLENLVAEAVQVNIPFNNPITGFCAFTHKAGIHAKAILANPSTYEILNPSDFGLSRYIHFANRLTGWNAIKSRVEQLNLNLSDDQVKEVTTKIKKLGDVRPLTIDDVDSIIKDFHADISTPLIQANDGPGPVDEEDSLLDNVKRPKLN*

BLASTP Hit 2:

Score Expect Method Identities Positives Gaps

689 bits(1778) 0.0 Compositional matrix adjust. 323/391(83%) 363/391(92%) 1/391(0%)

>jgi|Hanpo2|45133|e_gw1.1.228.1 cytosolic (T+WP)

MAQTNPYGPNPSDFLSNVSKFQIIDSTLREGEQFANAFFTLDMKLKIAKALDEFGVDYIELTSPVASEQAREEVEAICKLGLKTSKILTHIRCHMHDAKVAVETGVDGVNIFIGTSSFLRQHSHGKDMSYITKSAIEVIEFVKSKGVEVRFSTEDSFRSDIVDLLNIYSTVDKLGVNRIGIADTVGGANPRQVYELIRTIKSVVSCDIETHFHNDTGCAIANAYTALEAGAKYIDTCVLGIGERNGIVPLGGFMARMIVADPDYVKSKYQLKKIRDLENLVADIVQVNIPFNNPITGFCAFTHKAGIHAKAILANPSTYEILNPEDFGLSRYIHFANRLTGWNAIKSRVEQLNLKLSDDQIKEVTTKIKLMGDVRPLNIEDVDSIIKDYHADVSEEHANKRQKTN*

Browsed homocitrate synthase v1, identical to BLASTP Hit 1

>jgi|Hanpo2|17183|fgenesh1_kg.6_#_144_#_isotig04828

MPDQYQKVSEEQHKKVQVNPYGPNPSDYLSNVRSFQLIESTLREGEQFANAFFSTEKKIEIAKALDDFGVDYIELTSPVASEQSRIDCEAICKLGLKAKILTHIRCHMDDAKVAVETGVDGVDVVIGTSQFLRQYSHGKDMSYITKSAVEVIEFVKSKGIEIRFSSEDSFRSDIVDLLNIYKTVDKIGVNRVGIADTVGCANPRQVYELVKTLKSVVSCDIECHFHNDTGCAIANAYTALEAGAKLIDVSVLGIGERNGITPLGGLMARMIAADREYVMSKYKLHKLRDLENLVAEAVQVNIPFNNPITGFCAFTHKAGIHAKAILANPSTYEILNPSDFGLSRYIHFANRLTGWNAIKSRVEQLNLNLSDDQVKEVTTKIKKLGDVRPLTIDDVDSIIKDFHADISTPLIQANDGPGPVDEEDSLLDNVKRPKLN*

Browsed homocitrate synthase v2, identical to BLASTP Hit 2

>jgi|Hanpo2|84565|estExt_Genewise12.C_1_t20201

MAQTNPYGPNPSDFLSNVSKFQIIDSTLREGEQFANAFFTLDMKLKIAKALDEFGVDYIELTSPVASEQAREEVEAICKLGLKTSKILTHIRCHMHDAKVAVETGVDGVNIFIGTSSFLRQHSHGKDMSYITKSAIEVIEFVKSKGVEVRFSTEDSFRSDIVDLLNIYSTVDKLGVNRIGIADTVGGANPRQVYELIRTIKSVVSCDIETHFHNDTGCAIANAYTALEAGAKYIDTCVLGIGERNGIVPLGGFMARMIVADPDYVKSKYQLKKIRDLENLVADIVQVNIPFNNPITGFCAFTHKAGIHAKAILANPSTYEILNPEDFGLSRYIHFANRLTGWNAIKSRVEQLNLKLSDDQIKEVTTKIKLMGDVRPLNIEDVDSIIKDYHADVSEEHANKRQKTN*

>LYS20 YDL182W SGDID:S000002341

YG:nucleus/mitochondrial cytosolic (T+WP)

MTAAKPNPYAAKPGDYLSNVNNFQLIDSTLREGEQFANAFFDTEKKIEIARALDDFGVDYIELTSPVASEQSRKDCEAICKLGLKAKILTHIRCHMDDAKVAVETGVDGVDVVIGTSKFLRQYSHGKDMNYIAKSAVEVIEFVKSKGIEIRFSSEDSFRSDLVDLLNIYKTVDKIGVNRVGIADTVGCANPRQVYELIRTLKSVVSCDIECHFHNDTGCAIANAYTALEGGARLIDVSVLGIGERNGITPLGGLMARMIVAAPDYVKSKYKLHKIRDIENLVADAVEVNIPFNNPITGFCAFTHKAGIHAKAILANPSTYEILDPHDFGMKRYIHFANRLTGWNAIKARVDQLNLNLTDDQIKEVTAKIKKLGDVRSLNIDDVDSIIKNFHAEVSTPQVLSAKKNKKNDSDVPELATIPAAKRTKPSA*

BLASTP Hit 1, identical to BLASTP Hit 1 LYS21:

>jgi|Hanpo2|25221|fgenesh1_pm.6_#_193 cytosolic (T+WP)

MPDQYQKVSEEQHKKVQVNPYGPNPSDYLSNVRSFQLIESTLREGEQFANAFFSTEKKIEIAKALDDFGVDYIELTSPVASEQSRIDCEAICKLGLKAKILTHIRCHMDDAKVAVETGVDGVDVVIGTSQFLRQYSHGKDMSYITKSAVEVIEFVKSKGIEIRFSSEDSFRSDIVDLLNIYKTVDKIGVNRVGIADTVGCANPRQVYELVKTLKSVVSCDIECHFHNDTGCAIANAYTALEAGAKLIDVSVLGIGERNGITPLGGLMARMIAADREYVMSKYKLHKLRDLENLVAEAVQVNIPFNNPITGFCAFTHKAGIHAKAILANPSTYEILNPSDFGLSRYIHFANRLTGWNAIKSRVEQLNLNLSDDQVKEVTTKIKKLGDVRPLTIDDVDSIIKDFHADISTPLIQANDGPGPVDEEDSLLDNVKRPKLN*

BLASTP Hit 2, identical to BLASTP Hit 2 LYS21:

>jgi|Hanpo2|45133|e_gw1.1.228.1 cytosolic (T+WP)

MAQTNPYGPNPSDFLSNVSKFQIIDSTLREGEQFANAFFTLDMKLKIAKALDEFGVDYIELTSPVASEQAREEVEAICKLGLKTSKILTHIRCHMHDAKVAVETGVDGVNIFIGTSSFLRQHSHGKDMSYITKSAIEVIEFVKSKGVEVRFSTEDSFRSDIVDLLNIYSTVDKLGVNRIGIADTVGGANPRQVYELIRTIKSVVSCDIETHFHNDTGCAIANAYTALEAGAKYIDTCVLGIGERNGIVPLGGFMARMIVADPDYVKSKYQLKKIRDLENLVADIVQVNIPFNNPITGFCAFTHKAGIHAKAILANPSTYEILNPEDFGLSRYIHFANRLTGWNAIKSRVEQLNLKLSDDQIKEVTTKIKLMGDVRPLNIEDVDSIIKDYHADVSEEHANKRQKTN*

Alaine

Step from Pyruvate

SC yeast genome Alanine transaminase

SC yeastgenome Mitochondrial alanine transaminase

>ALT1 YLR089C SGDID:S000004079 mitochondrial (T+WP)

MLSLSAKNHFTVSNSITHVIKSYHIRTLTSSAEKMPHITTPFSTSASSTKLKAFRKVRPVLQRHSSSWIVAQNHRRSLSGQSSLNDLRHLNRFPHHTLKTSNNEFYPAEQLTLEDVNENVLKAKYAVRGAIPMRAEELKAQLEKDPQSLPFDRIINANIGNPQQLQQKPLTYYRQVLSLLQYPELLNQNEQQLVDSKLFKLDAIKRAKSLMEDIGGSVGAYSSSQGVEGIRKSVAEFITKRDEGEISYPEDIFLTAGASAAVNYLLSIFCRGPETGVLIPIPQYPLYTATLALNNSQALPYYLDENSGWSTNPEEIETVVKEAIQNEIKPTVLVVINPGNPTGAVLSPESIAQIFEVAAKYGTVVIADEVYQENIFPGTKFHSMKKILRHLQREHPGKFDNVQLASLHSTSKGVSGECGQRGGYMELTGFSHEMRQVILKLASISLCPVVTGQALVDLMVRPPVEGEESFESDQAERNSIHEKLITRAMTLYETFNSLEGIECQKPQGAMYLFPKIDLPFKAVQEARHLELTPDEFYCKKLLESTGICTVPGSGFGQEPGTYHLRTTFLAPGLEWIKKWESFHKEFFDQYRD*

BLASTP Hit 1:

Score Expect Method Identities Positives Gaps

615 bits(1587) 0.0 Compositional matrix adjust. 302/494(61%) 382/494(77%) 8/494(1%)

>jgi|Hanpo2|65785|estExt_Genewise1Plus.C_4_t20213 mitochondrial (T) unclear (WP)

MLRFRQMGIRNFSKQFAMPAAESVFKKQLSSTFEPASRLTASDLNPHALNAKYAVRGRIPTKAEELRNQLANESHSLPFNKIINANIGNPQQLDQKPLTFYRQVLALLQYPELMNHQAVVDVLPKDLIERARTLLDHIGSVGAYSHSQGVPYIRQRVADFISKRDGYESSPDDIFLTAGASTAVSYLLNLLSLGPQTGFLIPIPQYPLYTASLALNNSTALPYYLNEKDDWSINAEDLVKIIEDAQAQGVEARCLVLINPGNPTGAILKPEAIADLLTVAAEYGLVVIADEVYQENIFNGQFVSVKKVLKQLQEADGSGKFAEVQLASLHSTSKGISGECGQRGGYMELVGFEDSVRAQLLKLASISLCPPVSGQALVELMINPPTEGQESYPLYKQERDAIHEALKERSSLLYEAFNTMEGVTCQKPEGAMYLFPSLTLSKKVFEEAAKVGMEPDEYYCGQLLENTGICAVPGSGFGQVEGTWHVRTTFLAPGTEWIEDWKKFHAKFMEEHQ*

Browsed alanine transaminase; Identical to BLASTP Hit 1

>jgi|Hanpo2|13274|fgenesh1_pg.4_#_400

MPAAESVFKKQLSSTFEPASRLTASDLNPHALNAKYAVRGRIPTKAEELRNQLANESHSLPFNKIINANIGNPQQLDQKPLTFYRQVLALLQYPELMNHQAVVDVLPKDLIERARTLLDHIGSVGAYSHSQGVPYIRQRVADFISKRDGYESSPDDIFLTAGASTAVSYLLNLLSLGPQTGFLIPIPQYPLYTASLALNNSTALPYYLNEKDDWSINAEDLVKIIEDAQAQGVEARCLVLINPGNPTGAILKPEAIADLLTVAAEYGLVVIADEVYQENIFNGQFVSVKKVLKQLQEADGSGKFAEVQLASLHSTSKGISGECGQRGGYMELVGFEDSVRAQLLKLASISLCPPVSGQALVELMINPPTEGQESYPLYKQERDAIHEALKERSSLLYEAFNTMEGVTCQKPEGAMYLFPSLTLSKKVFEEAAKVGMEPDEYYCGQLLENTGICAVPGSGFGQVEGTWHVRTTFLAPGTEWIEDWKKFHAKFMEEHQ*

>ALT2 YDR111C SGDID:S000002518 cytosolic (T+WP)

MTMTHQQDLKGVFTAKDLDFKPAGKITKKDLNTGVTKAEYAVRGAIPTRADELKEELKKNPEVLPFDDIINANIGNPQQLDQKPLTFTRQVLAILEYPEILRVGHNELASLNLFSRDALERAERLLNDIGGSIGAYSHSQGVPGIRQTVADFITRRDGGEPATPEDIYLTTGASSAATSLLSLLCKDSQTGLLIPIPQYPLYTASASLFNAQVLPYYLDEESNWSTNSDEIEKVVQDALKKQIRPSVLIVINPGNPTGAVLSEETIARICLIAAKYGITIISDEVYQENIFNDVKFHSMKKVLRKLQHLYPGKFDNVQLASLHSISKGFMDECGQRGGYMEIIGFSQEIRDALFKLMSISICSVVTGQAVVDLMVKPPQPGDESYEQDHDERLKIFHEMRTRANLLYETFKELEGIECQKPQGAMYLFPRLVLPKKALCESERLGIEPDEFYCTSLLESTGICTVPGSGFGQRPGTYHVRTTFLAPGTKWIQDWKEFHQDFFSKYRN*

BLASTP Hit 1 identical to BLASTP Hit 1 ALT1:

>jgi|Hanpo2|57543|estExt_Genewise1.C_4_t20217 mitochondrial (T) unclear/M (WP)

MLRFRQMGIRNFSKQFAMPAAESVFKKQLSSTFEPASRLTASDLNPHALNAKYAVRGRIPTKAEELRNQLANESHSLPFNKIINANIGNPQQLDQKPLTFYRQVLALLQYPELMNHQAVVDVLPKDLIERARTLLDHIGSVGAYSHSQGVPYIRQRVADFISKRDGYESSPDDIFLTAGASTAVSYLLNLLSLGPQTGFLIPIPQYPLYTASLALNNSTALPYYLNEKDDWSINAEDLVKIIEDAQAQGVEARCLVLINPGNPTGAILKPEAIADLLTVAAEYGLVVIADEVYQENIFNGQFVSVKKVLKQLQEADGSGKFAEVQLASLHSTSKGISGECGQRGGYMELVGFEDSVRAQLLKLASISLCPPVSGQALVELMINPPTEGQESYPLYKQERDAIHEALKERSSLLYEAFNTMEGVTCQKPEGAMYLFPSLTLSKKVFEEAAKVGMEPDEYYCGQLLENTGICAVPGSGFGQVEGTWHVRTTFLAPGTEWIEDWKKFHAKFMEEHQ*
